# M6A-mediated upregulation of circMDK promotes tumorigenesis and acts as a nanotherapeutic target in hepatocellular carcinoma

**DOI:** 10.1186/s12943-022-01575-z

**Published:** 2022-05-06

**Authors:** Ashuai Du, Shiqin Li, Yuzheng Zhou, Cyrollah Disoma, Yujie Liao, Yongxing Zhang, Zongpeng Chen, Qinglong Yang, Pinjia Liu, Sixu Liu, Zijun Dong, Aroona Razzaq, Siyi Tao, Xuan Chen, Yuxin Liu, Lunan Xu, Qianjun Zhang, Shanni Li, Jian Peng, Zanxian Xia

**Affiliations:** 1 Department of Cell Biology, School of Life Sciences, Central South University, Tongzipo Road, Changsha, 410013 China; 2 Department of Infection Diseases, Guizhou Provincial People’s Hospital, Guizhou 550000 Guiyang, China; 3 Department of General Surgery, Guizhou Provincial People’s Hospital, Guizhou 550000 Guiyang, China; 4 Institute of Reproduction and Stem Cell Engineering, School of Basic Medical Science, Central South University, Changsha, 410078 China; 5 Department of Geriatric Surgery, Xiangya Hospital, Central South University, Changsha, 410008 China; 6 Hunan Key Laboratory of Animal Models for Human Diseases, Hunan Key Laboratory of Medical Genetics & Center for Medical Genetics, School of Life Sciences, Central South University, Changsha, 410013 China

**Keywords:** Hepatocellular carcinoma (HCC), circRNA, N6-methyladenosine (m6A), IGF2BP1, ATG16L1, Apoptosis, Poly (β-amino esters) (PAEs), Nanoparticles (NPs)

## Abstract

**Background:**

Emerging evidence suggest the critical role of circular RNAs (circRNAs) in disease development especially in various cancers. However, the oncogenic role of circRNAs in hepatocellular carcinoma (HCC) is still largely unknown.

**Methods:**

RNA sequencing was performed to identify significantly upregulated circRNAs in paired HCC tissues and non-tumor tissues. CCK-8 assay, colony formation, transwell, and xenograft mouse models were used to investigate the role of circRNAs in HCC proliferation and metastasis. Small interfering RNA (siRNA) was used to silence gene expression. RNA immunoprecipitation, biotin pull-down, RNA pull-down, luciferase reporter assay and western blot were used to explore the underlying molecular mechanisms.

**Results:**

*Hsa_circ_0095868*, derived from exon 5 of the MDK gene (named circMDK), was identified as a new oncogenic circRNA that was significantly upregulated in HCC. The upregulation of circMDK was associated with the modification of N6-methyladenosine (m^6^A) and poor survival in HCC patients. Mechanistically, circMDK sponged miR-346 and miR-874-3p to upregulate ATG16L1 (Autophagy Related 16 Like 1), resulting to the activation of PI3K/AKT/mTOR signaling pathway to promote cell proliferation, migration and invasion. Poly (β-amino esters) (PAEs) were synthesized to assist the delivery of circMDK siRNA (PAE-siRNA), which effectively inhibited tumor progression without obvious adverse effects in four liver tumor models including subcutaneous, metastatic, orthotopic and patient-derived xenograft (PDX) models.

**Conclusions:**

CircMDK could serve as a potential tumor biomarker that promotes the progression of HCC via the miR-346/874-3p-ATG16L1 axis. The PAE-based delivery of siRNA improved the stability and efficiency of siRNA targeting circMDK. The PAE-siRNA nanoparticles effectively inhibited HCC proliferation and metastasis in vivo. Our current findings offer a promising nanotherapeutic strategy for the treatment of HCC.

**Graphical Abstract:**

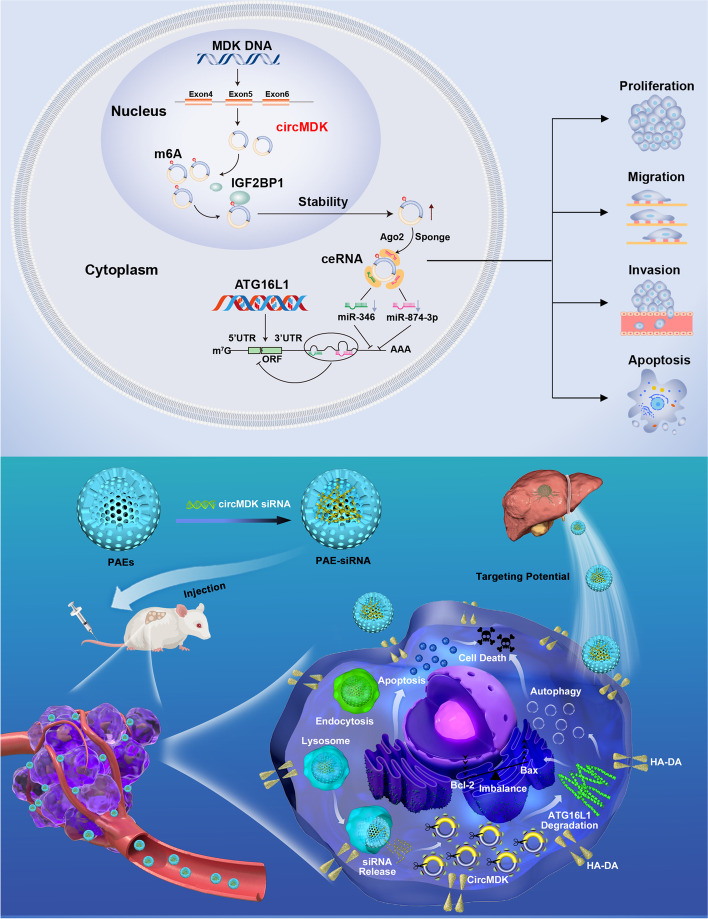

**Supplementary Information:**

The online version contains supplementary material available at 10.1186/s12943-022-01575-z.

## Introduction

Hepatocellular carcinoma (HCC) is the fifth most common cancer worldwide and the second leading cause of cancer death [1]. Despite rapid advances in diagnosis, surgical techniques, targeted therapies and immunotherapies, the 5-year overall survival rate of HCC patients remains unsatisfactory largely due to recurrent distant metastases and resistance to antitumor drugs. The underlying molecular mechanisms of HCC tumorigenesis, metastasis, and resistance still remain unclear. Therefore, it is essential to identify prognostic biomarkers, explore the potential mechanisms of HCC tumorigenesis and progression, and develop targeted therapies.

Circular RNAs (circRNAs) are another class of endogenous non-coding RNAs that are generated from introns or exons through back-splicing. They possess a covalently closed circular loop without 5’ end caps and 3’ polyadenylated tails [2]. Many studies have demonstrated that circRNAs can act as sponges for microRNAs (miRNAs) or bind to proteins, and altered circRNA levels can lead to aberrant expression of gene products [3]. An increasing number of studies have shown that circRNAs are widely expressed in mammals and are expressed to varying degrees in various cancers, such as esophageal squamous cell carcinoma, urothelial carcinoma, and colorectal cancer [4–6]. In addition, some circRNAs have been reported to participate in the development of HCC, regulating cell proliferation, tumor metastasis, and glycolysis [7–9]. Recent researches elucidated the role of some circRNA in HCC. For example, circASAP1 promoted HCC growth and pulmonary metastasis by enhancing the expression of MAPK1 and CSF-1 via sponging miR-326 and miR-532-5p [9], while circDLC1 inhibited HCC development and progression via circDLC1-HuR-MMP1 axis, serving as a potential therapeutic target for HCC [10]. CircRNA-SORE increases the sensitivity of HCC cells to sorafenib by inhibiting the Wnt/β-catenin pathway [11]. However, it remains to be explored whether HCC-associated circRNAs play a tumorigenic role through other regulatory mechanisms.

N6-methyadenosine (m6A) modification is emerging as a new frontier of epigenetic regulation with multiple functions including splicing, export, protein translation, stability and tumorigenesis [12], which has drawn increasingly more attention. m6A RNA methylation is the most prevalent internal mRNA modification in mammals and is mediated by m6A methyltransferases (METTL3/14, WTAP, RBM15/15B, KIAA1429, VIRMA and ZC3H13, called “writers”), demethylases (FTO, ALKBH5 and ALKBH3, called “erasers”) or m6A binding proteins (YTHDC1/2, YTHDF1/3, IGF2BP1/3, HNRNP and eIF3, called “readers”) [13]. Several members (e.g. METTL3, FTO and IGF2BPs) actively participate in diverse human cancers such as acute lymphoblastic leukemia [14], breast cancer [15] and endometrial cancer [16]. A bridge between m6A and circRNAs has been established earlier. For instance, m6A-mediated circRNAs that primarily localized to the cytoplasm resulted in enhanced of mRNA stability or increased of protein translation [17–19]. For instance, METTL3-dependent m6A was involved in the DGCR8-mediated maturation of miR126 and pri-miR126 [20]. In HCC cells, KIAA1429 could induce m6A methylation of GATA3, promoting malignant phenotypes [21]. Nevertheless, the roles of m6A-modified circRNAs in HCC still need further investigations.

In recent years, the remarkable progress in genome research has allowed gene therapy to emerge as a new approach to treating cancer. RNA interference (RNAi), a method of regulating target genes, shows promise for the developing novel molecular therapeutic drugs that encode “non-drug” targets which are not suitable for conventional therapies. RNAi molecules can be divided into miRNA, small interfering RNA (siRNA) and short hairpin RNA (shRNA) [22]. Among them, siRNAs show great potential for nucleic acid therapy because of their strong and specific RNAi triggering activity [23]. SiRNAs are short double-stranded RNAs that enable sequence-specific gene silencing of complementary messenger RNAs (mRNAs), induce mRNA degradation and inhibit the production of target proteins. The siRNA-based therapies have emerged as a promising strategy for targeting a variety of diseases [24]. However, the efficiency of gene silencing by naked siRNAs is very low because naked siRNA molecules are rapidly degraded by nucleases in the blood and undergo rapid renal clearance *in vivo * [25]. In addition, the large size and negative charge of siRNA impede its penetration into cell membranes and prevent its accumulation within cells. Therefore, effective delivery is a critical issue in bringing siRNA to target cells and tissues. To deliver siRNA efficiently, various materials have been developed, including lipids, polymers, dendrimers, polymer micelles, and metal-core nanoparticles (NPs). Interestingly, poly (β-amino esters) (PAEs) offer prospects to circumvent the difficulty in effective delivery of siRNAs. PAEs are a significant class of biodegradable synthetic polymers that can be degraded by intracellular esterase, resulting in significantly improved biocompatibility [26]. They have been investigated as gene and drug delivery vehicles and tissue engineering scaffolds considering their pH sensitivities, high biocompatibilities and structural diversity for flexible synthesis and gene loading.

In this study, we identified circMDK as an oncogenic circRNA in HCC by RNA sequencing (RNA-seq), and validated ATG16L1 as its target gene by sponging miR-346 and miR-874-3p, resulting in activation of PI3K/AKT/mTOR pathway. PAEs-mediated in vivo delivery of circMDK siRNA (PAE-siRNA) resulted in significant attenuation of tumor progression in four mouse models of HCC. Taken together, our study demonstrated that circMDK might serve as a potential biomarker for HCC, and provided a promising nanotherapeutic strategy for the treatment of HCC.

## Material and methods

### Patients and specimens

A total of 10 paired HCC and matched adjacent noncancerous liver (ANL) tissues were collected from patients who had undergone primary surgical resection in Mengchao Hepatobiliary Hospital of Fujian Medical University (Fujian, China). Another 35 paired HCC and ANL tissues were obtained from surgical resections of HCC patients without preoperative treatment at Guizhou Provincial People’s Hospital (Guizhou, China). All samples were identified by two pathologists independently and the human specimen collection was approved by the Ethics Committee of Mengchao Hepatobiliary Hospital and Guizhou Provincial People’s Hospital. Written informed consent was obtained from all patients according to the policies of the committee. The 10 paired samples were sent for RNA sequencing (RNA-seq) while the 35 paired samples were used for qRT-PCR and western blot analysis. All samples were stored at -80 °C until use. The detailed clinicopathological features are described in Supplementary Tables 1, 2 and 3.

## Statistical analysis

Data were assessed by SPSS 18.0 and expressed as mean ± SD. The statistical significance of differences was evaluated by two-tailed Student’s ttest or two-way ANOVA. Statistical analyses were performed using Prism software (GraphPad Software). The correlations among circMDK, IGF2BP1 and ATG16L1 expression in HCC patients were calculated by Pearson correlation analysis and *P* value computed using the *R* language function. *P* values < 0.05 were considered statistically significant.

## Results

### CircMDK is upregulated in HCC

**Fig. 1:**
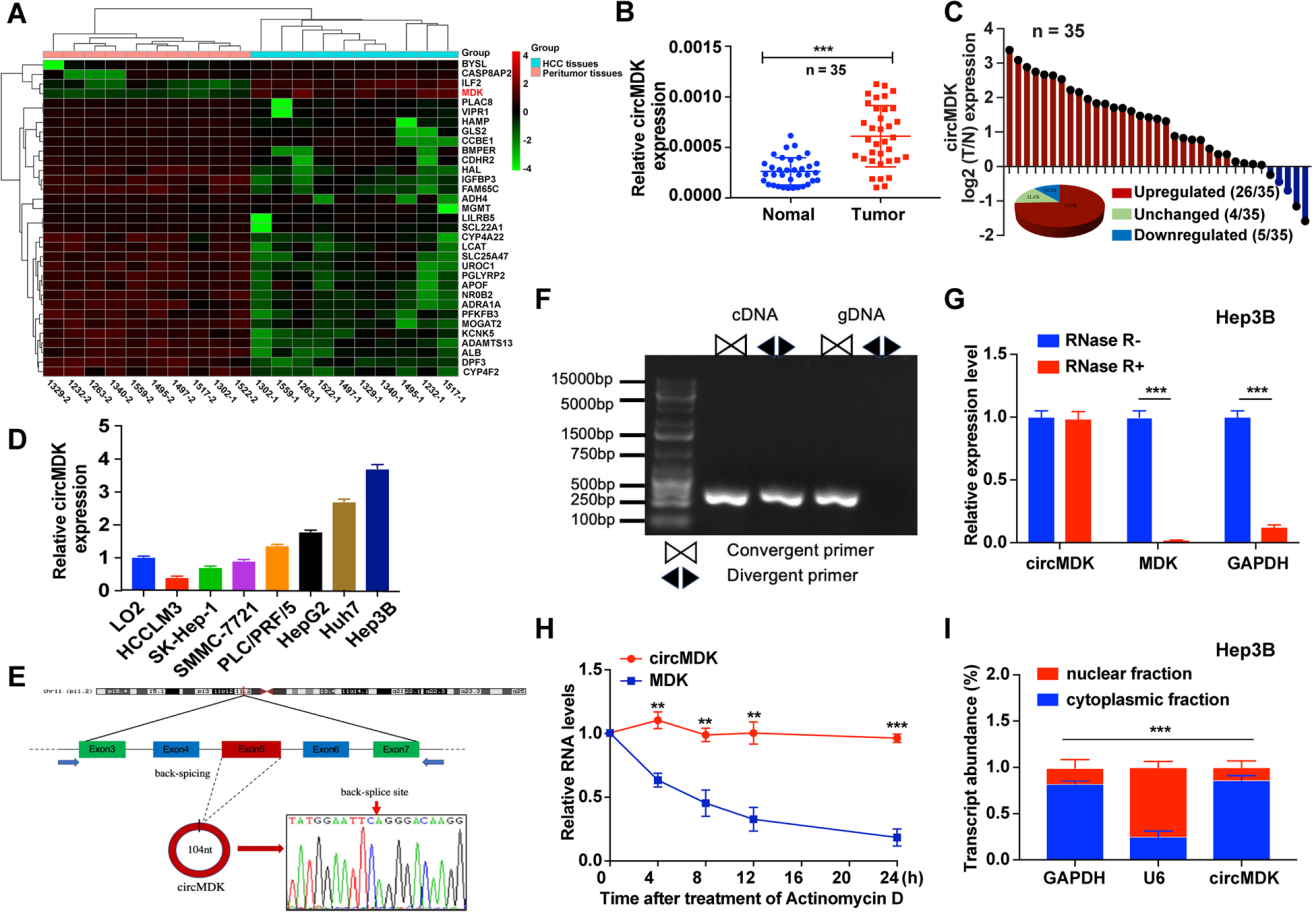
CircMDK expression profiling reveals that circMDK is upregulated in HCC. **A** Clustered heat map of the 42 dysregulated circRNAs in HCC tumor and peritumor tissues. The red and green strips represented high and low expression, respectively. **B** Analysis for RNA levels of circMDK in 35 paired samples of HCC were determined by qRT-PCR. The error bars represent standard deviation (SD) (*n* = 35). ***p < 0.001*.* **C** Histogram and pie chart of the proportions of HCC samples in which circMDK expression was upregulated (26/35, 74.3%, red), downregulated (4/35, 14.2%, blue), or no change (5/35, 11.4%, green), respectively. Log2 (T/N expression) value > 1 as significantly higher expression, < -1 as lower expression, and between -1 and 1 as no significant change. T, tumorous tissue; N, nontumorous tissue. **D** Analysis for RNA levels of circMDK in HCC cell lines (HCCLM3, SK-Hep-1, SMMC-7721, PLC/PRF/5, HepG2, Huh7 and Hep3B) and normal liver cell line (LO2). **E** The back-splice junction site of circMDK was identified by Sanger sequencing. **F** PCR analysis for circMDK and MDK in complementary DNA (cDNA) and genomic DNA (gDNA). **G** Analysis for RNA levels of circMDK and MDK after treated with RNase R in Hep3B cells. ***p < 0.001*.* **H** qRT-PCR for the abundance of circMDK and MDK mRNA in Huh7 cells treated with Actinomycin D at the indicated time points. **p < 0.01; ***p < 0.001*.* **I** Levels of circMDK in the nuclear and cytoplasmic fractions of Hep3B cells. ***p < 0.001*.* Data are shown as mean ± SD of three independent experiments

To investigate the role of circRNAs in HCC tumorigenesis, we performed RNA-seq analyses of ribosomal RNA-depleted total RNA obtained from 10 paired HCC and ANL tissues. A total of 92,204 distinct circRNAs were detected, of which four circRNAs were upregulated and 38 were downregulated in HCC compared with ANL tissues (Supplementary Fig. 1A, Fig. 1A). Based on differential expression levels of dysregulated circRNAs, we found that *hsa_circ_0095868* (termed circMDK in the remainder of the article) was significantly upregulated (fold change ≥ 10 and *p* < 0.05). To further verify our RNA-seq data, we detected the expression of circMDK in another 35 paired HCC samples by qRT-PCR (Fig. 1B). As shown in Fig. 1C, circMDK was significantly higher in 74.3% (26 of 35) of HCC tissues. The elevated expression of circMDK was significantly correlated with a higher TNM stage in HCC patients (Supplementary Table 3). We then analyzed the relationship between circMDK expression and prognosis of HCC patients in 35 paired HCC samples. Although the overall survival (OS) curve did not reach statistical significance (*p* > 0.05), the higher expression of circMDK conferred a poorer prognosis as compared to patients with lower circMDK expression. This lack of statistical significance could be due to the small sample size and short follow-up time; the 35 HCC patients were followed for only up to 30 months (Supplementary Fig. 1B). Moreover, the protein expression of MDK was higher in HCC tissues compared to normal liver tissues based on The Human Protein Atlas (THPA, https://www.proteinatlas.org) and its high expression was associated with a poorer 5-year survival probability (Supplementary Fig. 1C, D).

In addition, the expression levels of circMDK were confirmed in the normal liver cell line (LO2) and seven HCC cell lines including HCCLM3, SK-Hep-1, SMMC-7721, PLC/PRF/5, HepG2, Huh7 and Hep3B (Fig. 1D). Among the HCC cell lines, Hep3B and Huh7 cells showed the higher expression. Thus, we selected Hep3B and Huh7 cell lines to investigate the biological function and downstream regulatory mechanism of circMDK.

### Characteristics of circMDK in HCC cells

CircMDK is generated from exon 5 of MDK gene with a length of 104 nt. The back-spliced junction site of circMDK was amplified using divergent primers and confirmed by Sanger sequencing (Fig. 1E). PCR analysis showed that circMDK could be amplified by divergent primers in cDNA reverse-transcribed from random hexamers, but not gDNA primers (Fig. 1F). Resistance to digestion with RNase R, a highly processive 3’ to 5’ exoribonuclease that digests linear RNAs, confirmed that circMDK harbored a closed loop structure (Fig. 1G, Supplementary Fig. 1E). Treatment with Actinomycin D showed that the half-life of circMDK transcript within 24 h was more stable in comparison to MDK (Fig. 1H). The results of nuclear and cytoplasmic fractionation revealed that circMDK was predominantly localized in the cytoplasm (Fig. 1I, Supplementary Fig. 1F), which was consistent with the results of RNA FISH assay (Supplementary Fig. 1G). Collectively, these results demonstrated that circMDK was abundant, circular and stable transcript and was significantly up-regulated in HCC.

### Knockdown of circMDK impedes cell proliferation, migration and promotes cell apoptosis

**Fig. 2:**
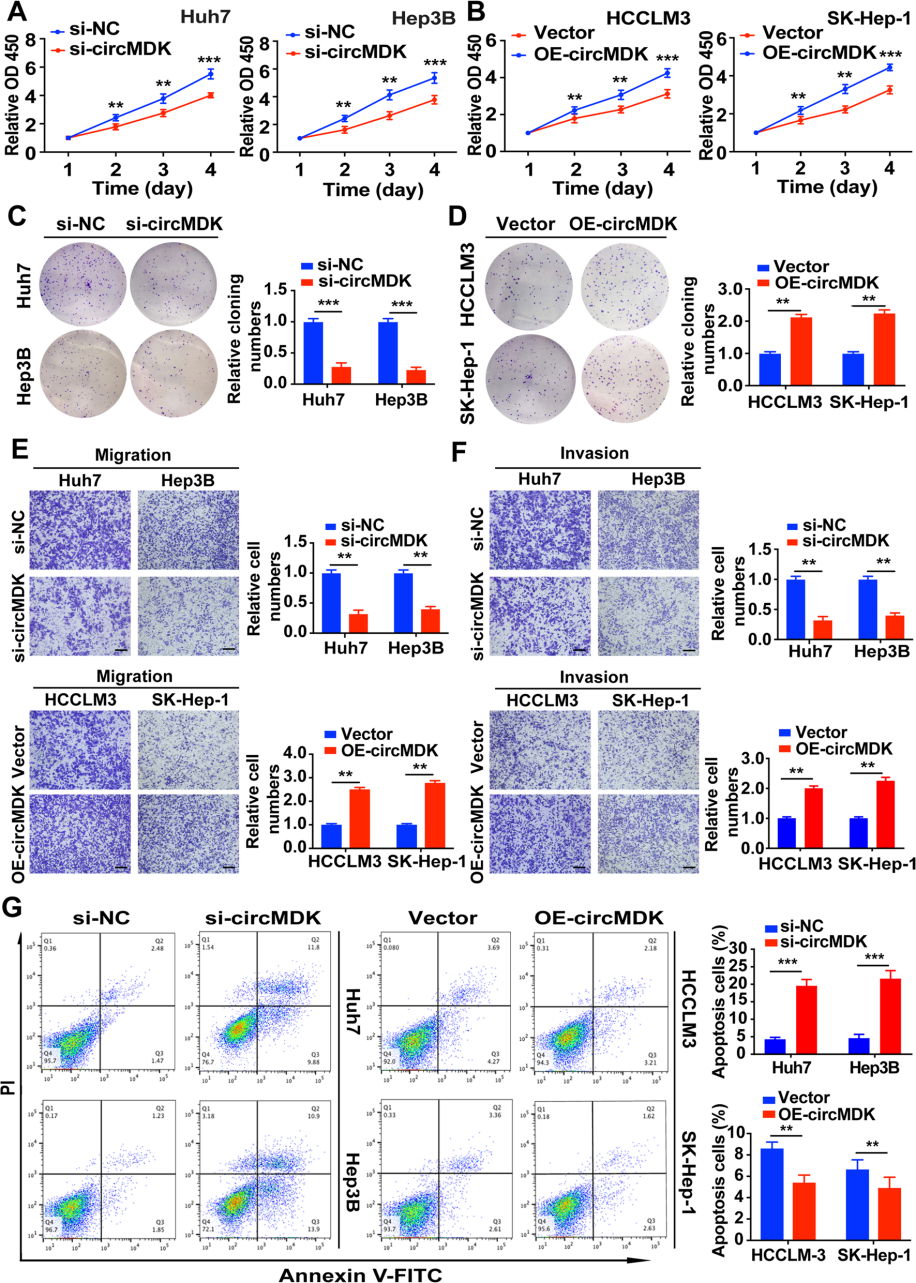
circMDK promotes cell proliferation, migration, invasion and impedes apoptosis in HCC cells. **A** Cell proliferation assays for Huh7 and Hep3B cells with silencing circMDK. **p < 0.01; ***p < 0.001*.* **B** Cell proliferation assays for HCCLM3 and SK-Hep-1 cells with overexpressing circMDK. **p < 0.01; ***p < 0.001*.* **C** Colony formation assays for Huh7 and Hep3B cells with silencing circMDK. ***p < 0.001*.* **D** Colony formation assays for HCCLM3 and SK-Hep-1 cells with overexpressing circMDK. **p < 0.01*.* **E** Cell migration analysis of Huh7 and Hep3B cells with silencing circMDK (top) and HCCLM3 and SK-Hep-1 cells with overexpressing circMDK (bottom). **p < 0.01*.* **F** Cell invasion analysis of Huh7 and Hep3B cells with silencing circMDK (top) and HCCLM3 and SK-Hep-1 cells with overexpressing circMDK (bottom). **p < 0.01*.* **G** Cell apoptosis analysis of Huh7 and Hep3B cells with silencing circMDK (left) and HCCLM3 and SK-Hep-1 cells with overexpressing circMDK (right). ***p < 0.001; **p < 0.01*.* Data are shown as mean ± SD of three independent experiments

To explore the role of circMDK in HCC progression in vitro, three siRNA specifically targeting the back-splicing region of circMDK (siRNA-#1, 2, 3) and overexpressing circMDK vector (OE-circMDK) were constructed to knockdown and overexpress circMDK. While siRNA-#1, 2, 3 all showed more than 50% knockdown efficiency in Huh7 and Hep3B cells, siRNA-#1 was chosen for the succeeding experiments due to its highest inhibitory effect (Supplementary Fig. 1H). Moreover, we found that siRNA-#1 could successfully knockdown circMDK expression but had no effect on mRNA expression of MDK in Hep3B cells (Supplementary Fig. 1I). CCK-8 assays and colony formation assays showed that silencing circMDK significantly reduced the proliferative capabilities of HCC cells while overexpressing circMDK promoted cell proliferation (Fig. 2A-D). Transwell assays showed that migration and invasion abilities of HCC cells were markedly reduced when circMDK was decreased (Fig. 2E). In contrast, increasing the level of circMDK increased the migration and invasion rate of cells (Fig. 2F). In addition, we also performed flow cytometry and found that knockdown of circMDK enhanced the apoptosis ratio of HCC cells while OE-circMDK led to opposite results (Fig. 2G). In summary, these results collectively indicated that silencing circMDK reduced the proliferation, migration and invasion, and promoted apoptosis in vitro.

### The m^6^A modification of circMDK improves the transcriptome stability

**Fig. 3:**
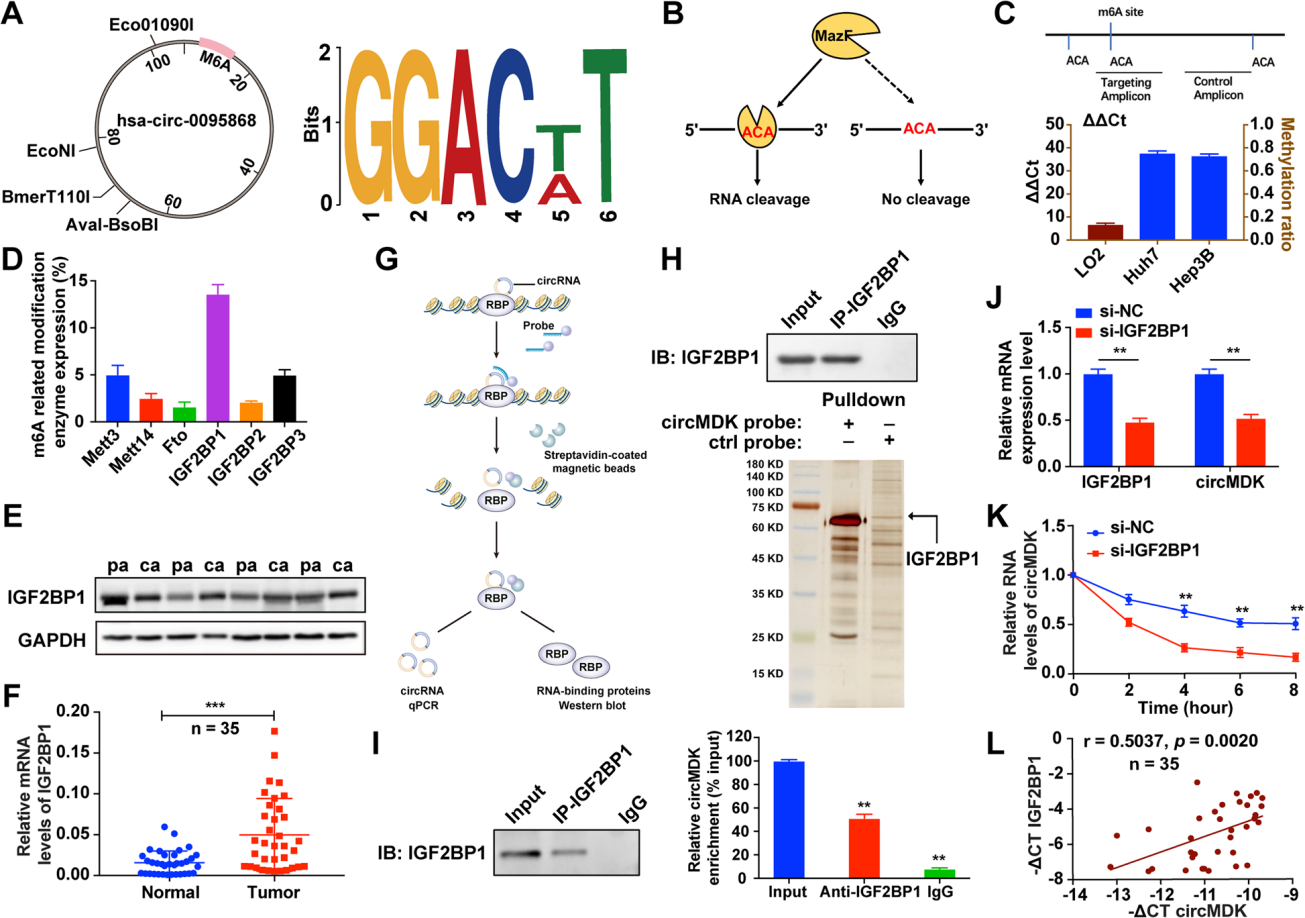
IGF2BP1 identifies m^6^A modified circMDK and improves the transcript stability of circMDK. **A** Bioinformatics analysis showed that there were m^6^A modification sites in circMDK. **B** The schematic diagram of MazF-PCR restriction endonuclease digestion. **C** The abundance of m^6^A modified circMDK in cells was detected by MazF-PCR. **D** The expression level of methylation-related proteins was verified by qRT-PCR. **E** The protein levels of IGF2BP1 in four paired HCC samples. pa: para-carcinoma tissues; ca: cancer tissues. **F** The qRT-PCR analysis of IGF2BP1 in 35 paired HCC samples. ***p < 0.001*.* **G** The schematic diagram of RIP assay. **H** Identification of the circMDK-protein complex pulled down by circMDK junction probe with protein extracts from Hep3B cells. The arrow indicates IGF2BP1. **I** RIP assays showing the association of IGF2BP1 with circMDK. Left, IP efficiency of IGF2BP1-antibody shown in western blotting. Right, relative enrichment representing RNA levels associated with IGF2BP1 relative to an input control. IgG antibody served as a control. **p < 0.01*.* **J** Transcript levels of IGF2BP1 and circMDK in negative control and si-IGF2BP1 HCC cells. **p < 0.01*.* **K** Reduction of circMDK RNA stability in IGF2BP1 knockdown HCC cells as compared with control. Cells were treated with Actinomycin D at the indicated time points. Error bars indicate SD. The *t* test was applied to analyze the statistical significance between two groups. **p < 0.01*.* **L** Correlation analysis revealed positive correlation between the levels of circMDK and IGF2BP1 mRNA in the tumorous tissues of the 35 HCC patients. Data are shown as mean ± SD of three independent experiments

Recent studies have suggested that m^6^A modification in mRNA and circRNAs is extremely widespread, and functionally modulates the eukaryotic transcriptome to influence RNA splicing, export, localization, translation and stability [27]. To explore whether circMDK undergoes m^6^A modification, we first predicted the m^6^A sites in circMDK using an online bioinformatics tool m^6^A Avar (http://m6avar.renlab.org/) and found one RRACU m^6^A sequence motif at exon 5 site of circMDK (Fig. 3A). Next, we performed MazF to provide nucleotide-resolution quantification of m^6^A methylation sites. MazF toxin, an ACA-sequence-specific endoribonuclease, is sensitive to m^6^A sites, representing the first m^6^A-sensitive RNA cleavage enzyme. The methyl-sensitivity of MazF allowed simple analyses of both m6A demethylase and methyltransferase activity (Fig. 3B). As shown in Fig. 3C, the m^6^A level was higher in Hep3B and Huh7 cells compared with LO2 cells. We then detected the mRNA expression of Mett3, Mett14, Fto, IGF2BP1, IGF2BP2 and IGF2BP3 in Hep3B cells, and found that the most abundantly expressed was IGF2BP1, suggesting the IGF2BP1 protein was a m6A-binding protein of circMDK (Fig. 3D). Additionally, the expression levels of IGF2BP1 were determined by western blot and qRT-PCR, which indicated that IGF2BP1 was highly expressed in HCC tissues compared with normal tissues (Fig. 3E and F).

To further investigate whether the interaction between circMDK and IGF2BP1 exerts a biological function, RNA pull-down assays were performed (Fig. 3G). As shown in Fig. 3H, we conducted a biotin-labeled RNA pull-down using a specific biotin-labeled circMDK probe (against the back-splice sequence) and a  control probe. The results of silver staining showed that circMDK and IGF2BP1 were prominently enriched in the circMDK probe group. Furthermore, the significant enrichment of circMDK was observed in IGF2BP1 immunoprecipitants by using the anti-IGF2BP1 antibody compared with the IgG pellet (Fig. 3I). In addition, we found that knockdown of IGF2BP1 resulted in the decreased expression of circMDK (Fig. 3J). After treatment with actinomycin D to block the newly synthesized circMDK RNA, silencing IGF2BP1 reduced the RNA stability of circMDK (Fig. 3K). The correlation analyses also suggested that the expression of circMDK was positively related to IGF2BP1 (Fig. 3L). In summary, these findings suggested that IGF2BP1 could bind to circMDK in vitro, and m^6^A modification enhanced the transcriptome stability of circMDK, which may partially account for the significant upregulation of circMDK in HCC.

### CircMDK acts as a sponge for miR-346 and miR-874-3p

**Fig. 4:**
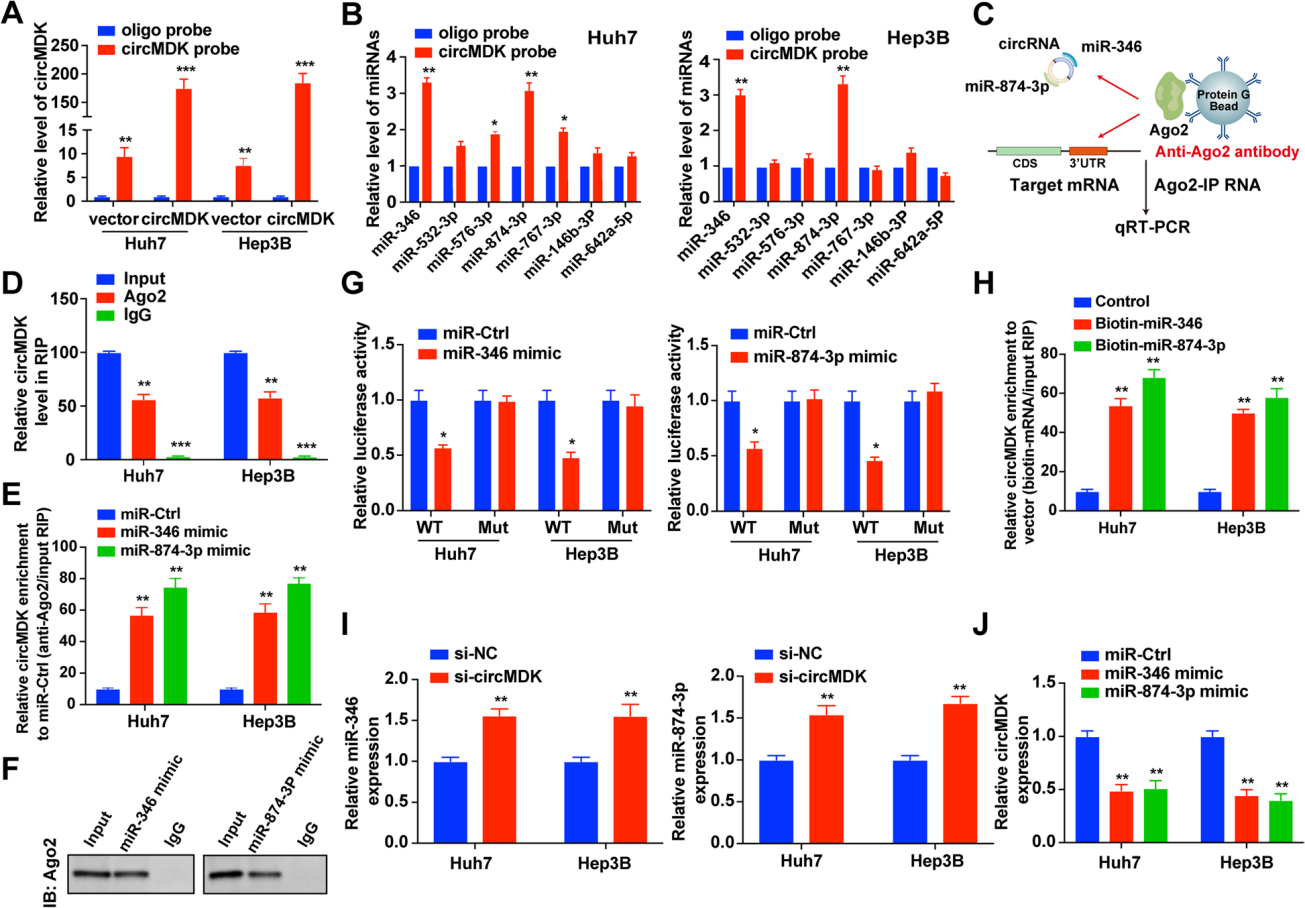
CircMDK acts as a ceRNA and sponges miR-346 and miR-874-3p. **A** Lysates from Huh7 and Hep3B cells with circMDK overexpression were subjected to biotinylated-circMDK pull-down assay and the expression levels of circMDK were measured by qRT-PCR. **p < 0.01; ***p < 0.001*.* **B** Relative expression of candidate miRNAs was quantified by qRT-PCR after the biotinylated-circMDK pull-down assay in HCC cells. *p < 0.05; **p < 0.01*.* **C** Schematic diagram of the Ago2-RIP assay. **D** Fold enrichment of circMDK in Huh7 and Hep3B cells. **p < 0.01; ***p < 0.001*.* **E** Enrichment of circMDK in Huh7 and Hep3B cells transfected with miR-346, miR-874-3p mimic, or miR-Ctrl. **p < 0.01*.* **F** Ago2 protein immunoprecipitated by Ago2 antibody or IgG was detected by western blot analysis. **G** Luciferase activity in HCC cells co-transfected with WT or mutant (346 Mut/874-3p Mut) circMDK plasmid together with miR-346 or miR-874-3p mimic or miR-Ctrl. *p < 0.05*.* **H** Enrichment of circMDK pulled down by biotin-miR-346, biotin-miR-874-3p, or control. **p < 0.01*.* **I** Relative levels of miR-346 and miR-874-3p in HCC cells transfected with si-circMDK or control. **p < 0.01*.* **J** Relative levels of circMDK in HCC cells transfected with miR-346, miR-874-3p mimics or miR-Ctrl. **p < 0.01*.* Data are shown as mean ± SD of three independent experiments. Ctrl, negative control; Mut, mutant type; WT, wild type

Considering the cytoplasmic distribution of circMDK, we hypothesized that circMDK might exert its effects by targeting miRNAs. According to the theory of competing endogenous RNA (ceRNA), circRNAs can share the same miRNAs with mRNA. Thus, we predicted the potential targets of circMDK using miRNA target prediction tools including miRDB, miRanda and TargetScan. We found that 17 candidate miRNAs were overlapped between three databases and selected the top 7 miRNAs for further analysis (Supplementary Fig. 2A). To verify our prediction, we designed a biotinylated-circMDK probe and confirmed the pull-down efficiency in HCC cells over-expressing circMDK (Fig. 4A). We found that miR-346 and miR-874-3p were abundantly pulled down by circMDK probe in Huh7 and Hep3B cells (Fig. 4B). To further consolidate the direct binding of miRNAs and circMDK, we conducted RIP assays using an anti-Ago2 antibody (Fig. 4C), which showed that circMDK was preferentially enriched in Ago2-RIPs compared with control IgG-RIPs (Fig. 4D). Furthermore, the Ago2-RIP assay also demonstrated that the enrichment of circMDK was much higher in miR-346 and miR-874-3p mimic groups than in the miR-Ctrl group (Fig. 4E and F). Subsequently, dual-luciferase reporter assays also showed that overexpression of miR-346 or miR-874-3p reduced the luciferase activity of the wildtype circMDK reporter gene (WT), but not the mutant circMDK vector (Mut) (Fig. 4G, Supplementary Fig. 2B). The results of FISH assay also confirmed that circMDK was colocalized with miR-346 and miR-874-3p in the cytoplasm (Supplementary Fig. 2C).

Moreover, biotin-labeled miRNA pulldown assays showed that the expression of circMDK was markedly elevated in HCC cells transfected with biotin-labeled miR-346 and miR-874-3p, compared with the control group (Fig. 4H). Meanwhile, circMDK silencing significantly increased the expression of miR-346 and miR-874-3p (Fig. 4I), whereas circMDK expression was obviously suppressed by overexpression of miR-346 or miR-874-3p (Fig. 4J). These results indicated that circMDK directly sponged miR-346 and miR-874-3p.

In addition, we investigated the involvement of miR-346 and miR-874-3p in the mechanism where circMDK promotes HCC development and progression. HCC cells were co-transfected with circMDK plasmids together with miR-346 or miR-874-3p mimics. We found that overexpression of miR-346 or miR-874-3p could inhibit cell growth and proliferation (Supplementary Fig. 3A, B), suppress migration and invasion (Supplementary Fig. 3C, D), and promote apoptosis (Supplementary Fig. 3E). These results suggested that the oncogenetic effects of circMDK were partially mediated by negative regulation of miR-346 and miR-874-3p.

### CircMDK decoys miR-346 and miR-874-3p to upregulate their common target, ATG16L1

**Fig. 5:**
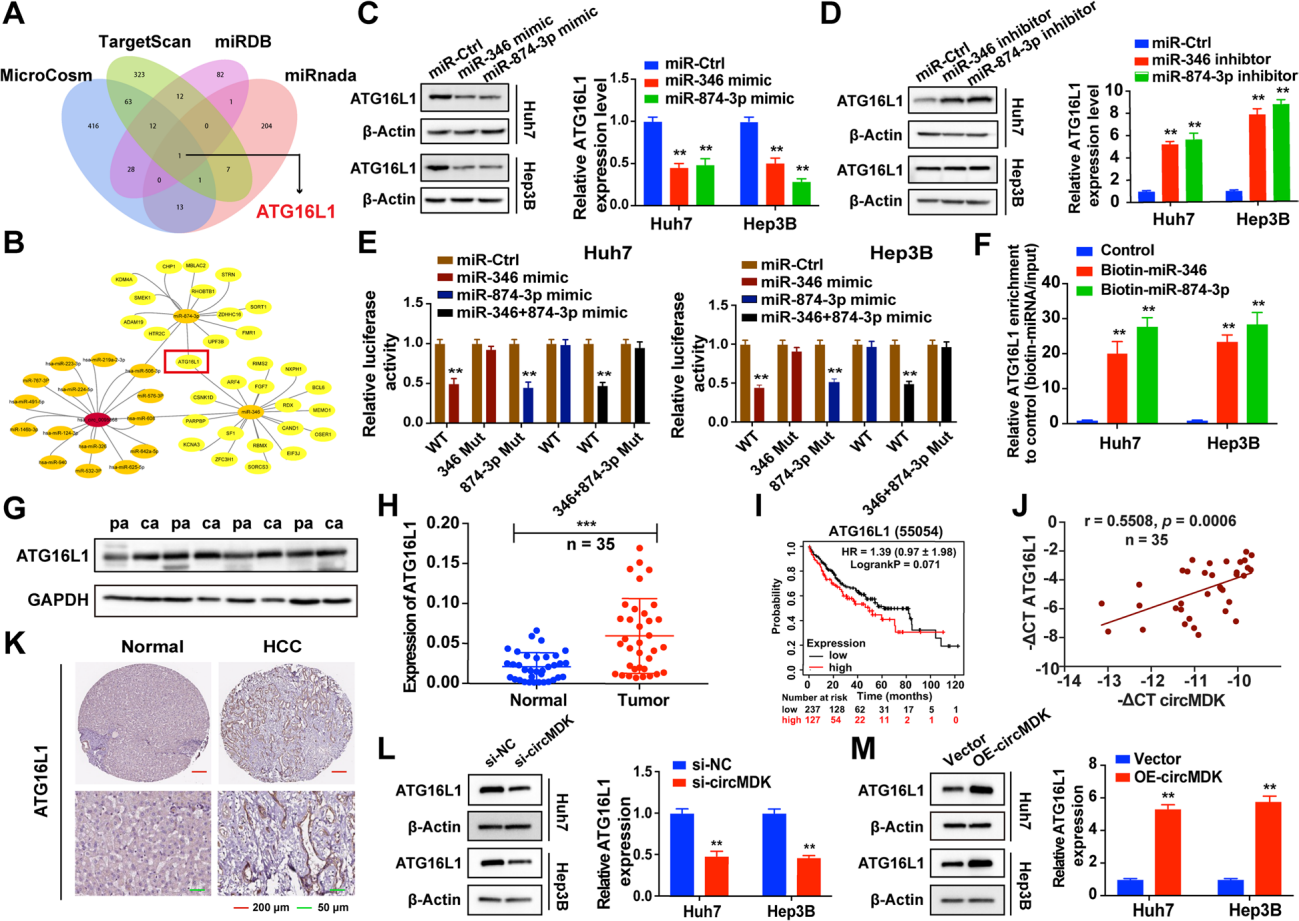
CircMDK functions as a decoy of miR-346 and miR-874-3p to increase ATG16L1 expression. **A** ceRNA analysis for circMDK by Cytoscape. **B** Schematic illustration of overlapping target mRNAs of miR-346 and miR-874-3p predicted by MicroCosm, TargetScan, miRDB and miRanda databases. **C** Relative protein (left) and mRNA (right) levels of ATG16L1 in HCC cells transfected with miR-Ctrl, miR-346 mimic, and miR-874-3p mimic. **p < 0.01. **D** Relative protein (left) and mRNA (right) levels of ATG16L1 in HCC cells transfected with miR-Ctrl, miR-346 inhibitor, and miR-874-3p inhibitor. **p < 0.01*.* **E** Luciferase assay in HCC cells co-transfected WT or mutant (346 Mut/874-3p Mut) ATG16L1 plasmid together with miR-346 and miR-874-3p mimic or miR-Ctrl. **p < 0.01*.* **F** Enrichment of ATG16L1 pulled down by biotin-labeled miR-346 and miR-874-3p, or negative control. **p < 0.01*.* **G** Relative protein levels of ATG16L1 in four paired HCC samples. pa: para-carcinoma tissues; ca: cancer tissues. **H** Relative mRNA levels of ATG16L1 in 35 paired HCC samples. mRNA and protein levels of ATG16L1 in four paired HCC samples. ***p < 0.001*.* **I** Kaplan–Meier analysis revealed the prognostic values of ATG16L1*.* **J** Correlation analysis revealed positive correlation between the levels of circMDK and ATG16L1 mRNA in the tumorous tissues of the 35 HCC patients. **K** The expression of ATG16L1 in human HCC tissue compared to normal liver tissue. **L** Relative protein (left) and mRNA (right) levels of ATG16L1 in HCC cells transfected with si-circMDK or si-NC. **p < 0.01*.* **M** Relative protein (left) and mRNA (right) levels of ATG16L1 in HCC cells transfected with circMDK overexpression or its empty vector. **p < 0.01*.* Data are shown as mean ± SD of three independent experiments

To further explore the regulatory role of miR-346 and miR-874-3p with circMDK, we first predicted the common target genes of miR-346 and miR-874-3p using MicroCosm, TargetScan, miRDB and miRanda (Fig. 5A). The intersection of these databases indicated ATG16L1 was the most possible target gene for circMDK (Fig. 5B). We found that the expression of ATG16L1 could be affected by modulating the expression of miR-346 and miR-874-3p (Fig. 5C and D). Luciferase reporter assays showed that overexpression of miR-346 and/or miR-874-3p repressed the luciferase activity in HCC cells transfected with the wildtype ATG16L1 3’-UTR reporter plasmid, whereas no obvious inhibition was observed in cells transfected with the corresponding mutant reporter plasmid (Fig. 5E). The predicted miRNA binding sites between ATG16L1 and miR-346/miR-874-3p were depicted in Supplementary Fig. 2D.

Moreover, biotin-labeled miRNA pulldown assays verified that ATG16L1 was the target gene of miR-346 and miR-874-3p (Fig. 5F). Further investigations showed that ATG16L1 was highly expressed in HCC tissues based on the results of western blot and qRT-PCR (Fig. 5G, H). Kaplan–Meier survival analysis was performed to report 120 months OS rates based on high or low ATG16L1 expression, and indicated that high expression of circMDK was associated with worse OS (Fig. 5I). We also found that there was a positive correlation between circMDK and ATG16L1 expression in HCC tissues (Fig. 5J). Subsequently, we used THPA (https://www.proteinatlas.org) proteomic data to verify the expression of ATG16L1 in human HCC tissues and normal liver tissues and the data showed that ATG16L1 was remarkably upregulated in HCC tissues (Fig. 5K). Further investigations showed that downregulation of circMDK decreased the expression of ATG16L, while its upregulation increased ATG16L expression both the mRNA and protein levels (Fig. 5L, M). Taken together, these findings indicated the existence of a circMDK-miR-346/miR-874-3p-ATG16L1 regulatory axis.

### ATG16L1 is responsible for circMDK-mediated cell proliferation, migration, invasion and apoptosis

**Fig. 6:**
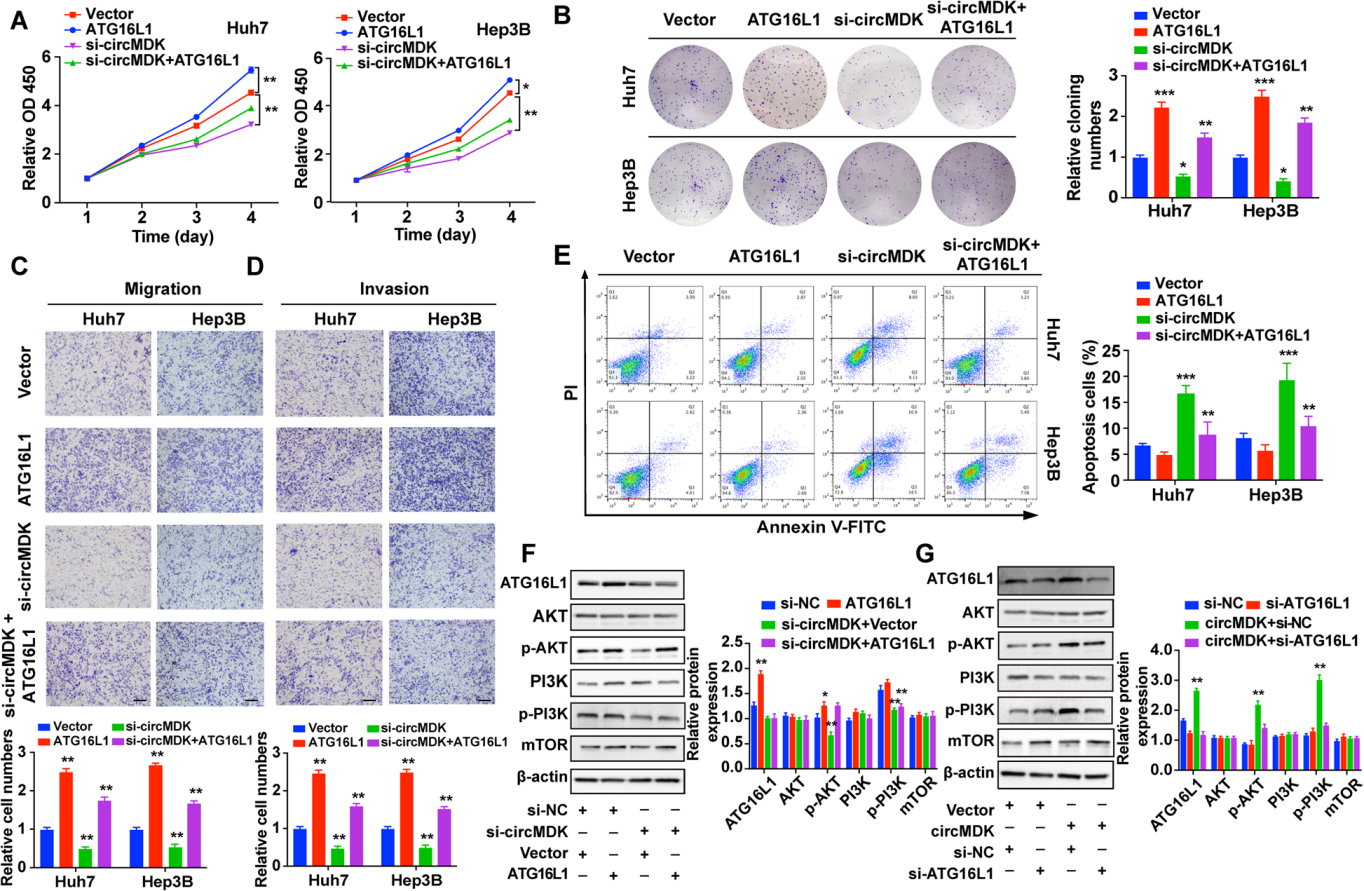
ATG16L1 is responsible for circMDK-mediated proliferation, migration, invasion and apoptosis. **A** Cell proliferation assay for HCC cells with ATG16L1 overexpression and circMDK knockdown. *p < 0.05; **p < 0.01*.* **B** Representative images (left) and quantification (right) of the colony formation assay in HCC cells. *p < 0.05; **p < 0.01; ***p < 0.001*.* **C** Representative images (top) and quantification (bottom) of migration assays in HCC cells. **p < 0.01*.* **D** Representative images (top) and quantification (bottom) of invasion assays in HCC cells. **p < 0.01*.* **E** Representative images (left) and quantification (right) of apoptosis assays in HCC cells. **p < 0.01; ***p < 0.001*.* **F** Expression changes (left) and quantification (right) of ATG16L1, AKT, p-AKT, PI3K, p-PI3K, mTOR in Hep3B cells co-transfected with si-circMDK together with ATG16L1 overexpression or (**G**) si-ATG16L1 together with circMDK overexpression. *p < 0.05; **p < 0.01. **H** Schematic diagram of circMDK-miR-346/miR-874-3p-ATG16L1 axis. Data are shown as mean ± SD of three independent experiments

To explore the ability of circMDK to promote tumor progression in an ATG16L1-dependent manner, Huh7 and Hep3B cells were transfected with si-circMDK or ATG16L1-overexpression vector. In vitro functional experiments demonstrated that the restoration of ATG16L1 expression partially rescued the regulatory effects of circMDK knockdown on proliferation (Fig. 6A and B), migration (Fig. 6C), invasion (Fig. 6D) and apoptosis (Fig. 6E) in HCC cells. ATG16L1 has been reported to promote tumorigenesis in ovarian cancer by activating the PI3K/AKT/mTOR pathway [28]. Therefore, we investigated whether modulating the circMDK-mir-346/miR-874-3p-ATG16L1 axis could activate PI3K/AKT/mTOR pathway. As shown in Fig. 6F, circMDK silencing decreased ATG16L1 protein levels and the phosphorylation levels of Akt (p-Akt) and PI3K (p-PI3K). In contrast, circMDK overexpression increased ATG16L1 protein levels and the levels of p-Akt and p-PI3K. In addition, the rescue experiments showed that ATG16L1 overexpression increased the levels of p-Akt and p-PI3K, whereas ATG16L1 knockdown had the opposite effects (Fig. 6G). Collectively, these findings demonstrated that circMDK was an oncogenic circRNA that promoted the cell growth, migration, invasion and inhibited apoptosis in HCC via the circMDK-mir-346/miR-874-3p-ATG16L1-PI3K/AKT/mTOR signaling pathway.

### Synthesis and characterization of PAE-siRNA complex in vitro

Considering the critical role of circMDK upregulation in HCC, we sought to develop an efficient delivery vehicle of siRNA targeting circMDK as a potential therapy for HCC. As shown in the previous results, siRNA delivery via Lipo3000 can effectively knock down the expression of circMDK and inhibit its effects on cell proliferation, migration and invasion ability in vitro. We then sought to further verify the in vivo effect of circMDK knockdown on the proliferation and metastasis of HCC.

**Fig. 7:**
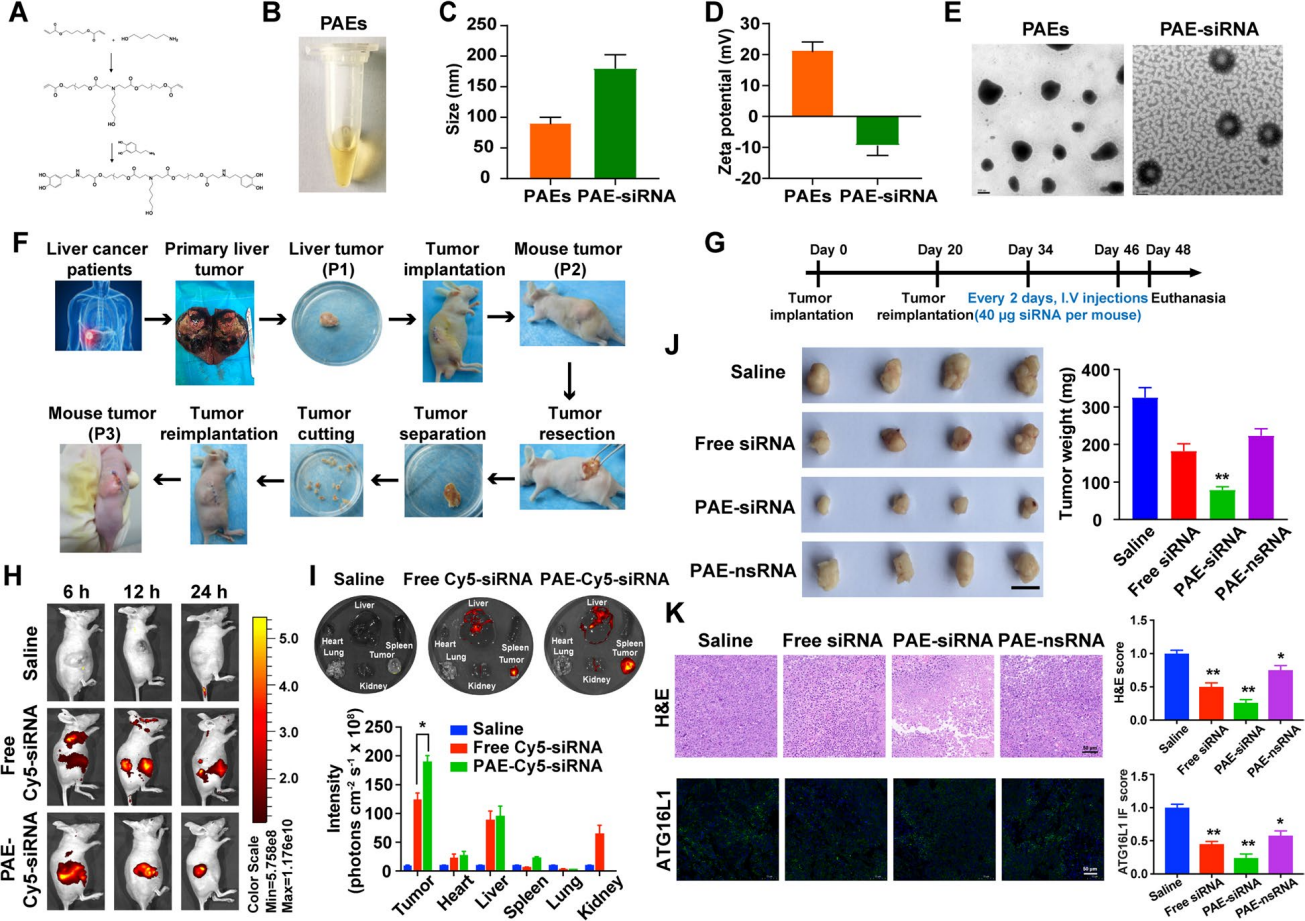
Characteristics and antitumor effects of PAE-siRNA complex in patient-derived xenograft (PDX) model. **A** Synthesis protocol and **B** appearance of positively charged PAEs nanoparticles. **C** Particle size of the PAEs and PAE-siRNA. **D** Zeta potential of the PAEs and PAE-siRNA. **E** TEM images of PAEs and PAE-siRNA. **F** Scheme of the establishment of PDX tumor model. Patient-derived primary liver tumors (P1) were cut into fragments with sizes of 3*3*3 mm and transplanted into the subcutaneous tissue of the Balb/c nude mice (P2). Then, tumor tissues of P2 were retransplanted into the subcutaneous tissue of the Balb/c nude mice (P3) using the same method. **G** Schematic illustration of PDX model establishment and treatment. **H** Biodistribution of PAE-siRNA complex in vivo. **I** Biodistribution of different Cy5-siRNA injections (*n* = 3) on major organs and tumors by ex vivo imaging after 24 h of treatment. *p < 0.05. **J** Representative images of the PDX tumors and tumor weight. Scale bar is 1 cm. **p < 0.01. **K** Representative images (left) and quantification (right) of H&E staining (top) and immunofluorescence (bottom) of ATG16L1. *p < 0.05; **p < 0.01*.* Scale bars are 50 µm

We synthesized a type of PAEs for targeted delivery of siRNA for circMDK. The branched PAEs were synthesized through Michael addition reaction of amines and acrylates using glycerol triacrylate as cross-linker (Fig. 7A). The obtained PAEs had a light-yellow appearance in solution (Fig. 7B) with a diameter of 92.57 nm and a positive zeta potential of 21.5 mV (Fig. 7C and D). Due to the protonation of amine groups derived positive charges in acidic environments, the circMDK siRNA adsorbed onto cationic PAEs via electrostatic interaction (Supplementary Fig. 4A). The morphology of the PAEs was further characterized by transmission electron microscope, which showed a well-defined core structure with uniformed size of ~ 100 nm (Fig. 7E). After binding of siRNA, the diameter of PAE-siRNA complex was increased to ~ 180 nm (Fig. 7C) and the zeta potential was reduced to -9.41 mV (Fig. 7D).

Furthermore, we explored the effect of PAE-siRNA complex on the stability of siRNA to serum nuclease by gel electrophoresis. Free siRNA and PAE-siRNA (100 nM siRNA equivalent) were incubated at 37℃ in DMEM medium with 10% FBS at multiple time points. As shown in Supplementary Fig. 4B, PAE-siRNA complex delayed the degradation of siRNA under FBS condition and the intensity of siRNA remained unchanged at least 6 h after incubation, suggesting that PAE-siRNA could protect naked siRNA from serum nuclease degradation. On the contrary, most of free siRNA rapidly degraded within 0.5 h in FBS incubation. This result indicated that PAE-siRNA complex effectively protected the siRNA against serum nuclease degradation.

To further study the cellular uptake of siRNA in vitro, we examined the internalization of free Cy5-siRNA and PAE-Cy5-siRNA (100 nM siRNA equivalent) in HepG2 cells. After incubating with different Cy5-siRNA formulations for 6 h, the PAE-Cy5-siRNA entered the HepG2 cells in much larger quantity than free Cy5-siRNA (Supplementary Fig. 4C), which suggested that PAE-siRNA complex could promote better internalization of siRNA and cellular uptake as compared to free siRNA.

### The effects of knockdown of circMDK with PAE-siRNA in vitro

Firstly, the cytotoxicity assay was employed to test the safety of PAE-siRNA complex. With increasing siRNA concentrations (from 0 to 5 µM), the PAE-siRNA complex did not exhibit apparent toxicity on HepG2 cells compared with Lipofectamine 3000 transfection reagent-mediated siRNA delivery (Lipo3000-siRNA group) (Supplementary Fig. 4D). Subsequently, we performed qRT-PCR to assess the knockdown effect of Lipo3000-siRNA, PAE-siRNA and PAE-nsRNA in HepG2 cells. As shown in Supplementary Fig. 4E, the PAE-siRNA (100 nM siRNA equivalent) had a remarkably stronger inhibitory effect on circMDK mRNA expression, which was downregulated by about 60% compared to the untreated control group.

Furthermore, we found that the proliferative capacity of HepG2 cells treated with PAE-siRNA was significantly reduced based on CCK-8 assay (Supplementary Fig. 4F). Similarly, PAE-siRNA complex impeded the colony-formation ability in comparison with the nontreated and PAE-nsRNA group in HepG2 cells (Supplementary Fig. 4G). The results from the transwell chamber system showed that PAE-siRNA complex suppressed the migration and invasion of HepG2 cells (Supplementary Fig. 4H). To sum up, these results suggested that PAE-siRNA complex inhibited cell growth, migration, and invasion in vitro.

### In vivo antitumor effects of PAE-siRNA complex

To explore the potential antitumor effects of PAE-siRNA complex in vivo, we constructed four HCC animal models, including subcutaneous model, metastatic model, PDX model, and orthotopic model.

### Antitumor effects and targeting potential in subcutaneous tumor model

To investigate the antitumor effects and biodistribution of PAE-siRNA in vivo, we constructed subcutaneous models in Balb/c nude mice. The general experimental procedure is illustrated in Supplementary Fig. 5A. We firstly investigated the in vivo antitumoral efficacy of PAE-siRNA on Balb/c nude mice bearing subcutaneous HepG2 tumor-bearing models. Briefly, HepG2 tumor-bearing mice were randomly divided into 4 groups with peritumoral injection of saline, free siRNA, PAE-siRNA, and PAE-nsRNA (4 µg siRNA per mouse equivalent, 15µL siRNA of 20 µM stock), respectively. Body weights of mice in all groups were not significantly affected by any treatment measures, indicating no significant toxicity (Supplementary Fig. 5B). As shown in Supplementary Fig. 5C, the tumor growth of PAE-siRNA group was significantly inhibited compared with saline control, and free siRNA injection also exhibited slight effects on tumor suppression. The tumor sizes and tumor weight in PAE-siRNA group were the smallest among four groups and the inhibitory efficiency reached about 60%, calculated by tumor weight (Supplementary Fig. 5D, E).

Analysis of the haematoxylin and eosin (H&E) tumor sections suggested that PAE-siRNA caused more necrosis than in other groups (Supplementary Fig. 5F). The tumor proliferation and malignancy were evaluated according to the expression of Ki-67. Visually, tumor cells in the PAE-siRNA treated group exhibited decreased Ki-67 expression (Supplementary Fig. 5G). IF staining of the tumor sections for ATG16L1 (green fluorescence) indicated that the decreased expression of ATG16L1 induced by PAE-siRNA was more obvious than the other groups (Supplementary Fig. 5H). Moreover, the TUNEL staining (green fluorescence, Supplementary Fig. 6A), Bax (red fluorescence, Supplementary Fig. 6B), Bcl-2 (yellow fluorescence, Supplementary Fig. 6C) and Caspase-3 staining (pink fluorescence, Supplementary Fig. 6D) of tumor tissue sections all showed that PAE-siRNA induced more apoptosis in most cells. These data in subcutaneous models demonstrated our hypothesis that circMDK silencing inhibits the progression of subcutaneous liver tumors.

To furtherly explore the biodistribution of PAE-siRNA in vivo, we intravenously injected free Cy5-siRNA and PAE-Cy5-siRNA (40 µg siRNA equivalent, 150µL siRNA of 20 µM stock) into Balb/c nude mice bearing subcutaneous HepG2 tumors respectively. After 6–24 h of injection, the PAE-Cy5-siRNA effectively accumulated into the tumor tissues as indicated by a higher degree of fluorescence imaging than the free siRNA (Supplementary Fig. 6E). After 24 h injection, major organs as well as tumor tissues were eviscerated for ex vivo imaging. We observed that mice treated with PAE-Cy5-siRNA complex exhibited higher level of fluorescence intensity at tumor sites than those treated with free Cy5-siRNA (Supplementary Fig. 6F). In addition, the PAE-siRNA was mainly eliminated by the liver and the kidneys (Supplementary Fig. 6F). It is consistent with previous report that the liver can rapidly uptake, degrade, and eliminate NPs, while the kidneys could excrete nanoparticles through urine [29, 30]. The tumor size and weights were also significantly suppressed by PAE-Cy5-siRNA complex (Supplementary Fig. 6G, H). Taken together, these results suggested that PAE-siRNA could passively accumulate into tumor tissues through the enhanced permeation and retention effect, thereby minimizing non-specific tissue distribution.

### Antitumor effects of PAE-siRNA complex in metastatic tumor model

We generated lung metastases models to explore the role of circMDK in regulating the progression of tumor metastasis in vivo (Supplementary Fig. 7A). No apparent change in body weight was observed during the period of treatment (Supplementary Fig. 7B).

After 2 weeks of tail vein injection of HepG2-luc cells, saline, free siRNA, PAE-siRNA and PAE-nsRNA (40 µg siRNA per mouse equivalent, 150µL siRNA of 20 µM stock) were injected into mice via tail vein, respectively. Subsequently, we obtained bioluminescence images (BLI) of mice to evaluate the tumor progression in vivo. As depicted in Supplementary Fig. 7C, the mice were imaged to visualize the lung metastases. We clearly observed that the reduced fluorescence intensity was weakest in PAE-siRNA group. Similarly, the results of BLI of lung and metastatic nodules in the lungs presented that PAE-siRNA complex decreased the number of metastatic lung nodules compared with the control group (Supplementary Fig. 7D, E). H&E staining of lung indicated that decreased tumor area was observed in the PAE-siRNA group than in other groups (Supplementary Fig. 7F). These results suggested that PAE-siRNA complex effectively suppressed tumor metastasis.

### Antitumor effects of PAE-siRNA complex in PDX model

As depicted in Fig. 7F, we constructed HCC PDX tumor models in Balb/c nude mice from primary liver tumor of two patients (P1) to subcutaneous tumor of mice (P2 and P3). In order to verify the accuracy of PDX model establishment, we performed H&E staining and immunohistochemical staining of Hep, a liver cancer specific marker. These results showed that PDX tumor model maintained the structural characteristics and a degree of similarity with the primary tumor (Supplementary Fig. 8A, B). The experimental procedure for the PDX model of HCC is depicted in Fig. 7G. There were no remarkable changes of mouse body weight during the whole treatment period, indicating no apparent systemic toxicity for all treatment groups (Supplementary Fig. 8C).

We also tested the biodistribution of PAE-siRNA at various time points after treatment of free Cy5-siRNA and PAE-Cy5-siRNA (40 µg siRNA per mouse equivalent, 150µL siRNA of 20 µM stock). The fluorescence imaging indicated that PAE-Cy5-siRNA exhibited a better targeting effect on tumor sites than free Cy5-siRNA (Fig. 7H). Furthermore, the results of biodistribution were also verified in ex vivo tissues at 24 h (Fig. 7I).

At the end of the intervention, we observed that PAE-siRNA group resulted in a more significant decrease in tumor sizes and weight than those from the saline control, free siRNA and PAE-nsRNA group (Fig. 7J). As shown in Fig. 7K, H, E analysis of the tumor sections showed increased necrotic area in the PAE-siRNA group. The expression of ATG16L1 detected by IF (green fluorescence) was the weakest in PAE-siRNA group, which indicated that the knockdown of circMDK by PAE-siRNA suppressed the tumor growth through decreasing the expression of ATG16L1 in PDX model.

### Antitumor effects of PAE-siRNA complex in orthotopic model

**Fig. 8:**
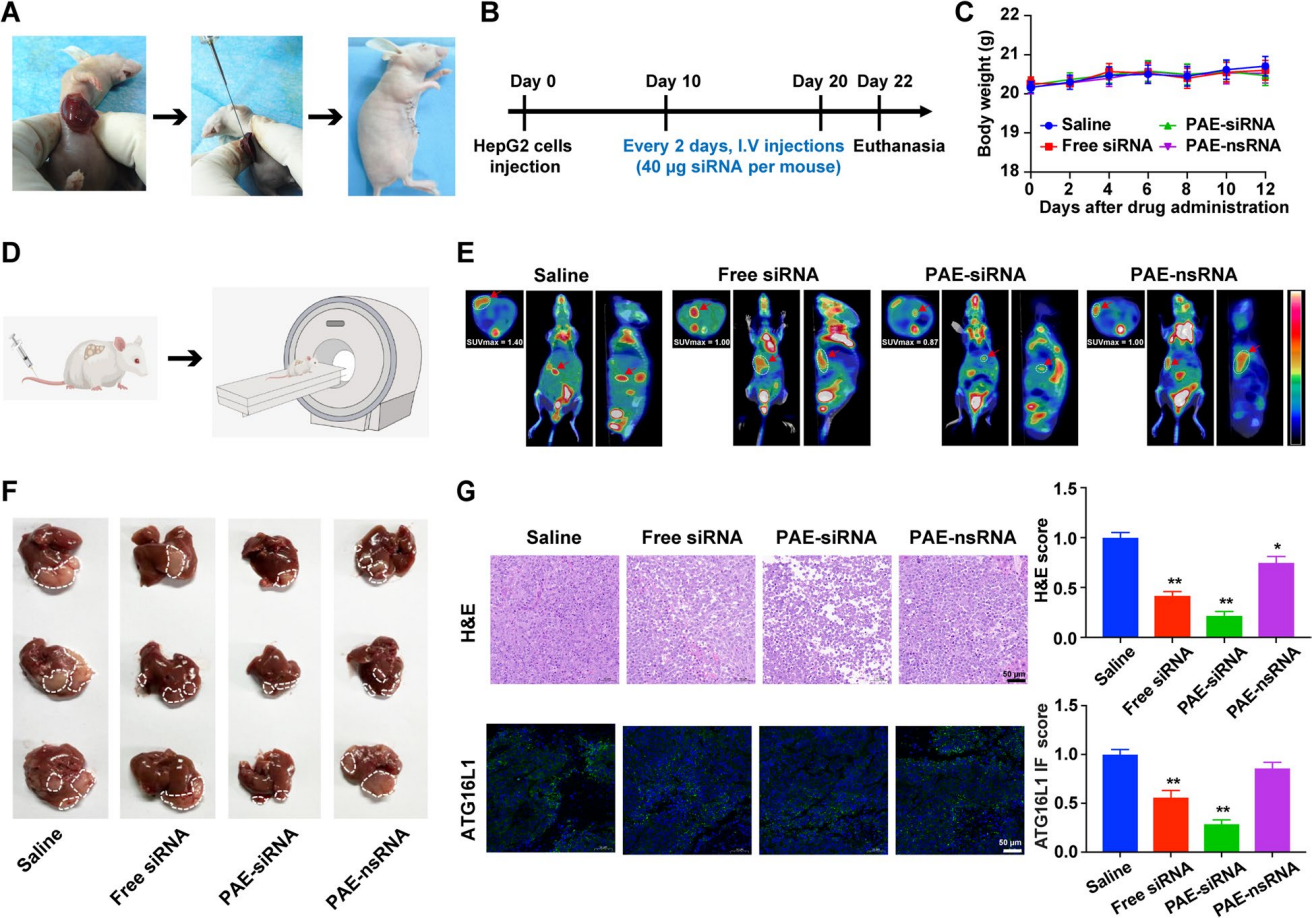
Antitumor effects of PAE-siRNA complex in orthotopic model. **A** Scheme of the establishment of orthotopic tumor model. HepG2 cells were injected into the lower lobe of liver of Balb/c nude mice to form orthotopic tumors. **B** Schematic illustration of orthotopic model establishment and treatment. **C** Changes of mouse body weight during experimental period. Data are shown as means ± SD (*n* = 6). **D** Schematic cartoon of PET/CT detection in mice. **E** Representative images of PET/CT scans from each group. Images of mice were acquired 24 h after intravenous injection of ^18^F-FDG. Shown from left to right are the axial, coronal, and lateral views. The white circles and red arrows indicated the regions of hepatic tumors. Standardized uptake values (SUV) were examined by ^18^F-FDG. **F** Representative images of the HepG2 orthotopic HCC tumors. The white dotted lines indicate tumor regions (*n* = 6). **G** Representative images (left) and quantification (right) of liver tumor sections stained with H&E (top) and immunofluorescence (bottom) of ATG16L1. *p < 0.05; **p < 0.01*.* Scale bars are 50 µm

To further reveal the potential therapeutic effect of PAE-siRNA for in vivo HCC, a more relevant animal model, the orthotopic model was employed to investigate the efficacy of antitumor. We established orthotopic hepatic tumor models to mimic tumor microenvironment in vivo (Fig. 8A), and evaluated the antitumor effect of siRNA by systemic and various drug administration (40 µg siRNA per mouse equivalent, 150µL siRNA of 20 µM stock, Fig. 8B). No significant change of mouse body weight was observed during the whole experimental period (Fig. 8C), indicating that there was no apparent systemic toxicity caused by different siRNA treatments.

To further investigate the antitumor effects of PAE-siRNA complex in orthotopic model, we performed PET/CT scans using ^18^F-FDG as a tumor metabolic marker of mice. Cartoon illustration of PET/CT scanning process was shown in Fig. 8D. As depicted in Fig. 8E, we can clearly see that there was relatively lower accumulation of ^18^F-FDG in the liver region marked by the arrows of mice treated with PAE-siRNA than in other groups. Similarly, we observed that PAE-siRNA complex significantly suppressed the liver growth of mice compared to the saline group (Fig. 8F). H&E staining analysis of the liver sections presented that more necrotic areas in the PAE-siRNA group than in other groups were observed (Fig. 8G). In addition, the IF of ATG16L1 (green fluorescence) was obviously decreased in PAE-siRNA group (Fig. 8G), suggesting that PAE-siRNA complex reduced the expression of ATG16L1.

### In vivo toxicity text of PAE-siRNA

To evaluate the toxicity and side effects of PAE-siRNA complex in vivo, the H&E-stained tissue slices of the major organs (heart, liver, spleen, lung and kidney) were analyzed after multiple dosing treatment. As shown in Supplementary Fig. 8D, no noticeable damage or pathological change were observed in the organ slices between the PAE-siRNA group and the control groups. In addition, there were no abnormal changes in ALT, AST, BUN, and CRE (Supplementary Table 5). These results revealed that PAE-siRNA possessed no evident side effects, and can be a safe agent for further in vivo applications.

## Discussion

The differential expression of various circRNAs has been reported in various diseases, especially in cancers. Their stability, abundance, conservation, and spatiotemporal specificity made them a hotspot of biomedical research in recent years [31]. In this study, we found that circMDK expression was significantly increased in HCC tissues and cell lines. The increased expression of circMDK resulted in a poor prognosis, suggesting a critical tumor-promoting effect. In contrast to linear MDK, circMDK showed stable expression in HCC cells and was mainly localized in the cytoplasm, indicating a role in post-transcriptional gene regulation. In vitro functional assays showed that knockdown of circMDK promoted cell apoptosis and hindered proliferation, migration and invasion of HCC. The difference in expression and function of circMDK may be related to the tissue specificity of circRNA, which suggests the potential of circMDK as a promising prognostic biomarker to guide the development of personalized therapies for HCC patients.

The m^6^A modification is widespread throughout the transcriptome, accounting for about 0.2% to 0.6% of total adenosine of cellular RNA [32]. M^6^A modification has been shown to participate in the development of various cancers. The fate of m^6^A-modified RNA depends on the function of the different proteins that identify them, which impact stability, translation efficiency, subcellular localization, and alternative polyadenylation [33–35]. For example, lncRNA GAS5-AS was reported to repress tumorigenesis and metastasis of cervical cancer by enhancing GAS5 stability and regulating m6A modifications of GAS5, which was dependent on ALKBH5 and YTHDF2. Furthermore, ALKBH5 and YTHDF2 are involved in the dynamic regulation of the balance between m6A methyltransferase and demethylase [36]. Here, we identified a predicted m6A site in circMDK by a series of experiments and found that IGF2BP1 could bind to circMDK in vitro. Our findings showed that m^6^A modification of circMDK improved its RNA stability, which might partially account for the upregulation of circMDK in HCC. In addition to m^6^A modification, there might exist other mechanisms that are involved in the elevation of circMDK, such as DNA methylation, histone modification, and miRNA dysregulation, which deserve further exploration.

To date, more and more miRNAs have been found to take vital roles in the pathogenesis and progression of cancers [37]. In this study, we identified circMDK as a sponge for miR-346 and miR-874-3p. MiR-346 has been reported to regulate a variety of cellular processes, including oncogenesis, inflammatory response and differentiation [38, 39]. In cervical cancer, miR-346 enhances Ago2 expression to regulate the activity of other miRNAs and migration and invasion [40]. It is reported that miR-874-3p expression was abnormally reduced in a variety of cancers, including ovarian cancer [41], colorectal cancer [42] and HCC [43]. Here, our findings revealed the significance of the interaction of circMDK and miR-346/miR-874-3p in HCC tumorigenesis.

In general, as a ceRNA, the function of circRNA depends on its target mRNAs. Using online databases, ATG16L1 was predicted as a common potential target of miR-346 and miR-874-3p, which was further confirmed by luciferase reporter and biotin-labeled miRNA pull-down assays. ATG16L1, a member of autophagy family, plays an important role in the occurrence and development of various diseases [44]. ATG16L1 could be targeted by miR-142-3p, a novel miRNA that regulates autophagy in human colonic epithelial cells, suggesting the role of miRNA-mediated regulation of ATG16L1 in intestinal inflammation and Crohn disease [45]. In our present study, we found that the expression of ATG16L1 was increased in HCC cells and tissues, implying that autophagy may promote HCC progression. Furthermore, multiple studies have reported that the classical PI3K/AKT/mTOR pathway plays a significant negative regulatory role in the formation of autophagosomes [46–48]. For instance, ATG16L1 was associated with tumor invasion and metastasis through activation of Akt signaling [49]. Here, we demonstrated that circMDK up-regulates the expression of ATG16L1 by adsorption of miR-346 and miR-874-3p to activate PI3K/AKT/mTOR signaling pathway, and ultimately promoted proliferation, migration and invasion of HCC cells, as well as inhibition of apoptosis.

Additionally, we developed an siRNA-based NPs delivery in this study. The PAE-siRNA complex downregulated circMDK expression through siRNA silencing and facilitated entry into cells without additional chemical modifications. Applying biocompatible PAEs for siRNA delivery is beneficial: they can increase siRNA stability in serum, and enhance siRNA uptake by cells and tumor accumulation. This may be due to the smaller size of PAEs and positively charged surface. To our knowledge, this is the first study on the delivery of circRNA with PAEs in HCC. PAE-siRNA complex effectively down-regulated circMDK expression in HCC cells and exhibited adequate anti-tumor effects in four animal models (subcutaneous model, orthotopic model, lung metastasis model and PDX model). Notably, PAEs also showed no detectable toxicity in vitro or in vivo. This suggests the feasibility and safety of using PAEs as carriers to deliver circMDK siRNA for antitumor therapy. However, the detailed regulatory mechanism of PAE-siRNA complex in cell proliferation, migration, and invasion remains to be investigated in future works.

While we have revealed the oncogenic role of circMDK and its molecular mechanisms in HCC both in vitro and in vivo, there are several limitations that have to be discussed. First, while we have demonstrated the binding of circMDK with IGF2BP1 in vitro, our data do not further reveal the effect of inhibition of circMDK adenosine methylation on IGF2BP1 recruitment in this paper. For instance, how inhibiting the adenosine methylation of circMDK using a global inhibitor (e.g., Sinefungin) or by CRISPR technology could affect IGF2BP1 expression requires further investigations. In addition, the interaction between IGF2BP1 and miR-346/-874-3p was also not addressed in this study. Third, while we report here the overexpression of ATG16L1 in HCC cells and tissues, it is yet to be further verified by transmission electron microscopy or other assays whether the formation of autophagic vesicles is an important attribute in HCC tumorigenesis and whether such vesicles are formed following administration of our PAE-siRNA complex.

In conclusion, we demonstrated circMDK as an oncogenic circRNA and played an important role in tumorigenesis and progression of HCC by circMDK-miR-346/miR-874-3p-ATG16L1 axis. Our study is the first to demonstrate a direct and effective approach to suppressing HCC by PAE-siRNA complex targeting circRNAs, which suggests a promising nanotherapeutic option for HCC.

## Supplementary Information

**Additional file 1:** Supplementary materials and methods. References.** Supplementary Table 1.** Clinical information of 10 HCC patient samples used for RNA-sequencing.** Supplementary Table 2.** Clinical information of 35 HCC patients’ samples for validation of circMDK expression.** Supplementary Table 3.** Associations between the expression levels of circMDK and the clinicopathological characteristics of 35 HCC patients.** Supplementary Table 4.** Primers, siRNA, shRNA sequences and antibodies.** Supplementary Table 5.** Blood biochemistry of tumor-bearing mice treatedwith saline, PAE-nsRNA and PAE-siRNA. Abbreviations.** Supplementary Figure 1.** The correlation characterization of circMDK, MDK expression, and prognosis in HCC, (A, B, C, D, E, F, G).** Supplementary Figure 2.** The binding site of miR-346 and miR-874-3p with circMDK, (A, B, C, D).** Supplementary Figure 3.** MiR-346 and miR-874-3p are responsible for circMDK-mediated proliferation, migration, invasion and apoptosis, (A, B, C, D, E).** Supplementary Figure 4.** Characterization and effects of knockdown of circMDK with PAE-siRNA complex in vitro, (A, B, C, D, E, F, G, H).** Supplementary Figure 5.** The antitumor effects of PAE-siRNA complex in subcutaneous hepatic tumors, (A, B, C, D, E, F, G, H).** Supplementary Figure 6.** Biodistribution of PAE-siRNA complex in subcutaneous tumor model, (A, B, C, D, E, F, G, H).** Supplementary Figure 7.** Antitumor effects of PAE-siRNA complex in metastatic tumor model, (A, B, C, D, E).** Supplementary Figure 8.** Validation diagrams for PDX model construction and Histological observation of tissue sections, (A, B, C, D).

## Data Availability

All data generated or analyzed during this study are included in this published article and its Additional files.

## Abbreviations

ANL: Adjacent noncancerous liver
BLI: Bioluminescence images
circRNAs: Circular RNAs
HCC: Hepatocellular carcinoma
H&E: Haematoxylin and eosin
miRNAs: microRNAs
m^6^A: N6-methyladenosine
PAEs: poly (β-amino esters)
PDX: patient-derived xenograft
RNAi: RNA interference
RNA-seq: RNA sequencing
shRNA: short hairpin RNA
siRNA: small interfering RNA
