# Supplementary material for: M6A-mediated upregulation of circMDK promotes tumorigenesis and acts as a nanotherapeutic target in hepatocellular carcinoma
Source: Mol Cancer. 2022 May 6;21:109. doi: 10.1186/s12943-022-01575-z (PMC9074191; doi:10.1186/s12943-022-01575-z)
Supplement: Supplementary file 1 — Additional file 1: Supplementary materials and methods. References. Supplementary Table 1. Clinical information of 10 HCC patient samples used for RNA-sequencing. Supplementary Table 2. Clinical information of 35 HCC patients’ samples for validation of circMDK expression. Supplementary Table 3. Associations between the expression levels of circMDK and the clinicopathological characteristics of 35 HCC patients. Supplementary Table 4. Primers, siRNA, shRNA sequences and antibodies. Supplementary Table 5. Blood biochemistry of tumor-bearing mice treatedwith saline, PAE-nsRNA and PAE-siRNA. Abbreviations. Supplementary Figure 1. The correlation characterization of circMDK, MDK expression, and prognosis in HCC, (A, B, C, D, E, F, G). Supplementary Figure 2. The binding site of miR-346 and miR-874-3p with circMDK, (A, B, C, D). Supplementary Figure 3. MiR-346 and miR-874-3p are responsible for circMDK-mediated proliferation, migration, invasion and apoptosis, (A, B, C, D, E). Supplementary Figure 4. Characterization and effects of knockdown of circMDK with PAE-siRNA complex in vitro, (A, B, C, D, E, F, G, H). Supplementary Figure 5. The antitumor effects of PAE-siRNA complex in subcutaneous hepatic tumors, (A, B, C, D, E, F, G, H). Supplementary Figure 6. Biodistribution of PAE-siRNA complex in subcutaneous tumor model, (A, B, C, D, E, F, G, H). Supplementary Figure 7. Antitumor effects of PAE-siRNA complex in metastatic tumor model, (A, B, C, D, E). Supplementary Figure 8. Validation diagrams for PDX model construction and Histological observation of tissue sections, (A, B, C, D). [file 12943_2022_1575_MOESM1_ESM.docx]

**Supplementary Materials and Methods**

**CircRNAs sequencing and annotation**

Total RNA from HCC and adjacent noncancerous liver (ANL) tissues were extracted using TRIzol reagent (TransGen Biotech, Beijing, China) according to the manufacturer’s instructions. Ribosomal RNA (rRNA) was removed with a Ribo-Zero Magnetic kit (Epicentre, Madison, WI, USA) and linear RNA was digested by RNase R (Epicentre) before construction of the RNA-seq libraries. Briefly, rRNA and linear RNA depleted samples were fragmented and subjected to complementary DNA (cDNA) synthesis with random hexamer primers. After repairing the ends and adding adapters, the ligated cDNA products were purified and subjected to 13-16 cycles of PCR amplification. High-throughput sequencing was then performed by HiSeq 3000 (Illumina, San Diego, CA, USA) with a 150-bp paired-end run and evaluated the sequencing quality of all sequencing reads. Quantile normalization and subsequent data processing were performed using the *R* software package. Significant differential expressed circRNAs were screened by fold change > 2 or < −2 and *p* value < 0.05.

**Cell lines and cell culture**

The normal human liver cell line (LO2), and human HCC cell line (HCCLM3, SK-Hep-1, SMMC-7721, PLC/PRF/5, HepG2, HepG2-luc, Huh7, Hep3B) were all purchased from the Chinese Cell Bank of the Chinese Academy of Sciences (Shanghai, China). All cells were maintained and stored following the instructions obtained from their providers. Briefly, LO2, HCCLM3, SK-Hep-1, SMMC-7721, PLC/PRF/5, HepG2, HepG2-luc, Huh7 and Hep3B were maintained in Dulbecco’s modified eagle medium (HyClone, Logan, UT, USA). All culture mediums contained 10% fetal bovine serum (FBS) (Gibco, USA) and 1% penicillin and streptomycin (Gibco, USA). All cell lines were cultured in a humidified incubator containing 5% CO_2_ at 37 °C.

**Nucleic acid electrophoresis**

The cDNA and genomic DNA (gDNA) PCR products were resolved using 2% agarose gel electrophoresis with TAE running buffer. DNA was separated by electrophoresis at 120 V for 30 min. The DNA marker was Marker L (50-500 bp) (Sango Biotech, China). The bands were visualized by UV irradiation.

**Total RNA extraction and qRT‐PCR**

Total RNA was extracted using TRIzol Reagent (Invitrogen, Carlsbad, USA), and the concentration and purity of total RNA were determined by an ultraviolet spectrophotometer (Eppendorf, Hamburg, Germany). Reverse transcriptions were performed using the PrimeScript RT Master Mix (Takara, Japan) with random primers. For miRNAs, reverse transcriptions were performed using the PrimeScript RT Reagent Kit (Takara, Japan) with specific stem-loop primers. The cDNA amplification was performed using SYBR Green SuperMix (Roche, Basel, Switzerland) and ABI 7900HT Fast Real-Time PCR system (Applied Biosystems, CA, USA). GAPDH and U6 were used as internal reference genes. The results were analyzed by the 2^-ΔΔCt^ method. All primer sequences are listed in Supplementary Table 4.

**Plasmid construction and cell transfection**

To overexpress circMDK, circMDK cDNA was inserted into the pLO-ciR (BioSune, Jinan, China). Amplified from the cDNA of HCC cells, full length ATG16L1 was cloned into the pcDNA3.1 (+) vector, while the full length of 3’UTR of ATG16L1 was inserted into the pmirGLO vector. PmirGLO-mut (circMDK), pmirGLO-mut (ATG16L1 3’UTR), miRNA mimics, miRNA inhibitors, ATG16L1 siRNA and circMDK siRNA were synthesized by Ribobio (Guangzhou, China). For transient transfection of plasmids, siRNAs, miRNA mimics and the miRNA inhibitors were transfected into cells using Lipofectamine 3000 reagent (Invitrogen, Waltham, MA, USA). All cell transfections were based on the manufacturer’s instructions. The sequences of these nucleic acids are listed in Supplementary Table 4.

**Actinomycin D and RNase R treatment.**

HCC cells were planted into six-well plates. Up to 60% confluency after 24 h, cells were treated with 5 μg/mL Actinomycin D or DMSO and collected at indicated time points. The RNAs (10μg) from HCC cells were treated with 5 U/μg RNase R (Epicentre Technologies) and incubated for 30 min at 37 °C. Then, the treated RNAs were reverse transcribed with divergent primer or convergent primer and detected by qRT-PCR assay and PCR assay followed by nucleic acid electrophoresis.

**Cell Counting Kit-8 (****CCK-8) and colony formation assays**

For the cell proliferation assay, 1 × 10^3^ cells were seeded in 100 μL of complete culture media in 96-well plates for various time points. CCK-8 assay (Beyotime, Shanghai, China) was performed to measure cell viability according to manufacturer’s instructions. The cells were subsequently incubated at 37°C for 2 hours, and the absorbance was measured at 450 nm by a microplate reader (Eppendorf, Germany). For the colony formation assay, 300 cells were inoculated into 6-well plates and cultured at 37°C for 14 days. The cells were then fixed with 4% paraformaldehyde (PFA) and stained with 0.1% crystal violet (Beyotime, Shanghai, China).

**Transwell migration and invasion assay**

Transwell assay was carried out as previously described. Briefly, the HCC cells were seeded in the upper chamber with serum-free DMEM and the lower chamber was filled with culture medium with 10% FBS as an attractant. After 48 h, the cells in the Matrigel (Sigma-Aldrich, USA) were fixed by 4% PFA and stained with 0.1% crystal violet (Beyotime, Shanghai, China). The cells were observed and photographed under light microscope, and 10 fields were selected to count the cells to reflect cell mobility. Triplicate independent experiments were performed.

**Flow cytometry (FCM) assay for cell apoptosis**

The Annexin V-FITC/Propidium iodide (PI) double staining kit (Vazyme, Nanjing, China) was employed to examine cell apoptosis in keeping with the protocol provided by the manufacturer. as previously described^1^,Briefly, the cells were harvested and stained with Annexin V-FITC and PI respectively, the FCM (BD FORTESSA, USA) was employed to measure cell apoptosis ratio. The FITC was detected at 530 nm, and PI was detected at 575 nm, respectively.

**Luciferase reporter assay**

The sequences of circMDK and ATG16L1-3’UTR and their corresponding mutation were designed, synthesized and inserted into p-GLO Dual-Luciferase vector (Vigenebio, Maryland, USA), as previously described^2^. Briefly, the luciferase reporter plasmids were co-transfected into HCC cells with miR-346 or miR-874-3p mimics or the negative control. The relative luciferase activity was measured with the Dual-Luciferase Reporter Assay System (Promega, USA) according to the manufacturer’s instructions.

**RNA** **fluorescence in situ hybridization (****FISH)**

Cy3-labeled oligonucleotide probe for circMDK and FAM-labeled oligonucleotide probe for miR-346 and miR-874-3p were applied for RNA FISH, as previously described^3^. Briefly, miR-346 and miR-874-3p were designed and synthesized by Ribobio (Guangzhou, China). The hybridization was performed in HCC cells as previously reported. Image acquisition was performed by a Zeiss LSM 900 confocal microscope (Carl Zeiss) with Zeiss Efficient Navigation (ZEN) software (Carl Zeiss).

**Biotin-labeled miRNA pulldown assay**

Cells were transfected with biotin-labeled miR-346 or miR-874-3p or miR-Ctrl (50 nmol/L), and then cell lysates were harvested 48 hours later. Simultaneously, streptavidin-Dyna beads (Invitrogen) were coated with yeast tRNA (Invitrogen) and incubated with rotation at 4°C for 2 hours. Then the beads were washed with splitting buffer and resuspended with lysis buffer. Sample lysates were mixed with precoated beads and incubated overnight at 4°C on a rotator. Beads were then pelleted to remove unbound materials and washed 6 times with ice-cold lysis buffer. RNA was isolated and subjected to qRT-PCR analysis.

**RNA-binding protein immunoprecipitation (RIP)**

RIP assay was performed by using a Magna RIP RNA-Binding Protein Immunoprecipitation Kit (Millipore), as previously described^4^, Briefly, Hep3B cells were harvested after 48 h post transfection and lysed in RIP lysis buffer on ice for 30 min. After centrifugation, the supernatant was incubated with 30 μL of Protein-A/G agarose beads (Roche, USA) and antibodies. IgG was used as a negative control. After overnight incubation, the immune complexes were centrifuged then washed six times with washing buffer. The beads-bound proteins were further analyzed using western blot. The immunoprecipitated RNA was applied to qRT-PCR analysis.

**RNA pulldown and silver staining**

The pull-down assay with biotinylated RNA was performed as described^5^. In brief, the biotin-coupled RNA complex was pulled down by incubating the cell lysates with streptavidin-coated magnetic beads (Invitrogen, Carlsbad, USA) following the manufacturer’s instructions. The enrichment of circMDK or IGF2BP1 in the capture fractions was evaluated by qRT-PCR analysis. The bound proteins were eluted from the packed beads and analyzed by SDS-PAGE. Silver staining was performed using the Fast Silver Stain Kit (Beyotime, Shanghai, China), as the protocol described. The probe sequences were shown as below: circMDK junction probe: 5’-GCCCUGCCUUGUCCCUAUGGGGCCUGGCCCACGCCCUCCCUC-3’; Control probe (ordered from Sangon Biotech, Shanghai, China): 5’-UUGUACUACACAAAAGUACUG-3’. The proteins in the capture complex were identified by western blot and silver staining analysis.

**Protein extraction and western blot**

Protein extraction and western blot were performed as previously described^6^. Antibodies were as follows: ATG16L1 (1:1,000; Abcam), IGF2BP1 (1:1,000; Proteintech), Ago2 (1:1,000; Proteintech), Akt (1:1,000; CST), p-Akt (Ser-473; 1:1,000; CST), PI3K (1:1,000; CST), p-PI3K (1:1,000; CST), mTOR (1:1,000; CST), β-Actin (1:5,000; Origene) and GAPDH (1:5,000; Origene).

**Materials**

Acryloyl chloride, glycerol, 5-amino-1-pentanol and 1,3-diaminopropane were purchased from Shanghai Sun-shine Chemical Technology, Co., Ltd. (Shanghai, China). All other solvents were analytical grade and obtained from Sinopharm Chemical Reagent Co., Ltd (Beijing, China) unless otherwise noted. PBS with pH 6.0 was used as the general solvent throughout the study. The synthesis procedure and samples were performed by Xiangya College of Pharmacy, Central South University (Changsha, China).

**Preparation of PAEs**

To synthesize PAEs, 1,4-butanediol diacrylate (5.0 g, 25.2 mmol), glycerol triacrylate (256.2 mg, 1.0 mmol) and 5-amino-1-pentanol (2.2 g, 21.0 mmol) were mixed in glass vial and kept stirring vigorously at 90℃ for 24 h in the dark firstly. Then acrylate- terminated poly (β-amino esters) was dissolved in DMSO to 500 mg/mL (160 μL), followed by adding 640 μL 1,3-Diaminopropane solution (0.25 M in DMSO) for 24 h incubation at room temperature with vigorously shaking. To collect PAEs, the final reaction mixture was added to 8 mL diethyl ether for 20 min incubation to precipitate completely. The final product of PAEs were collected by washing twice with diethyl ether and dried under reduced pressure.

**Preparation of PAE-siRNA complex.**

The positively charged PAEs (1ug/mL) were mixed with siRNA solution in PBS with pH 6.0, and shaken on a bench-top shaker for 1 h to complete the binding of siRNA onto the PAEs via electrostatic interaction. The siRNA was added into the PAEs solution in different concentrations to determine the saturated concentration of siRNA solution, with the weight ratio of siRNA to PAEs 50:1. The prepared samples were abbreviated as PAE-siRNA. The PAEs binding with nsRNA were abbreviated as PAE-nsRNA and served as control siRNA.

**Characterization of PAE-siRNA complex.**

The morphology of PAEs and PAE-siRNA complex was detected with a transmission electron microscope (TEM, Tecnai G2 F20, USA). The particle size and zeta potential values of PAEs and PAE-siRNA complex were respectively determined with a Zetasizer Nano ZS (Malvern Nano series, Malvern, UK).

For the stability assay against serum degradation, free siRNA and PAE-siRNA complex (100 nM siRNA equivalent) were incubated with 10% FBS at 37 °C. The sample was collected at each time point (0.5, 1, 2, 4, 6, and 24 h) for electrophoresis analysis.

**Cellular uptake of PAE-siRNA**

To study the cellular uptake of siRNA, we examined the internalization of different siRNA formulations into HepG2 cells. For visualization, circMDK siRNA was labelled with a Cy5 dye at the 5’-end of the sense strand (Cy5-siRNA) (Ribobio, Guangzhou, China). Different Cy5-siRNA formulations were incubated with HepG2 cells in the presence of serum for 6h. The cells were washed with PBS, fixed in 4% PFA solution for 15min, counterstained with DAPI and imaged with confocal microscopy using a 633nm laser excitation (Carl Zeiss LSM900).

***In vitro* cytotoxicity assays**

HepG2 cells were inoculated into 96-well plates at a density of 5 × 10^3^ cells/well. When the cells were 50% confluent, the cells were treated with PAE-siRNA or Lipofectamine 3000 transfection reagent-mediated siRNA delivery (Lipo3000-siRNA) at different concentrations from 0 to 5,000 μM. After 6 h, the medium was replaced by 100 μL complete medium, followed by 24 h incubation at 37 °C. Subsequently, 10 μL of CCK-8 was added to each well for an additional 2 h of incubation. Absorbance was then detected at 450 nm by a microplate reader (Eppendorf, Germany).

***In vivo* studies**

**Animals**

Balb/c nude mice (4-6 weeks, female) were obtained from Hunan SJA Laboratory Animal Co., Ltd. (Changsha, China) and raised in a specific pathogen-free environment. All animal studies were approved by the Department of Animal Research, Central South University. The animal studies were not blinded. All animals were included in the analysis.

**Biodistribution of PAE-siRNA complex**

We used Cy5-siRNA to minimize the interference from autofluorescence in the *in vivo* imaging studies. To study the tumor-targeting ability of PAE-siRNA complex, Balb/c nude mice with subcutaneous tumors were injected with saline, free Cy5-siRNA and PAE-Cy5-siRNA (40 µg Cy5-siRNA per mouse equivalent, 150 µL of 20 µM stock) through tail vein, respectively. At 6, 12 and 24 h post injection, the fluorescent images of the mice were imaged with IVIS Spectrum *in vivo* imaging system (PerkinElmer). After 24 h, mice were killed, the major organs and tumors were dissected, and the *ex vivo* fluorescent images were acquired using the same system.

**Antitumor effects *in vivo***

We conducted four liver tumor models, including subcutaneous tumor model, metastatic tumor model, patient-derived xenograft (PDX) tumor model, and orthotopic tumor model to investigate antitumor effects of the PAE-siRNA complex.

**Subcutaneous tumor model**

We injected 1×10^6^ HepG2 cells into the flank of Balb/c nude mice to form subcutaneous tumors. When the tumor sizes reached about 5 mm in diameter, mice were sorted to give nearly identical mean tumor sizes, and different siRNA formulations (4 µg siRNA per mouse equivalent, 15 µL of 20 µM stock) were injected as close as possible to the tumors (n = 6 mice per group). Saline was injected as the control group. The injections were repeated every 2 days for six injections. Tumor volume was calculated as follows: V (volume) = (length × width^2^)/2. On killing of animals, the tumor xenografts were excised, weighted and imaged.

**Pulmonary metastatic tumor model**

We injected 1×10^6^ HepG2-luc cells into Balb/c nude mice via tail vein for the establishments of pulmonary metastatic model. Two weeks after the tumor implantation, the mice were randomly divided into different groups: saline; free siRNA; PAE-siRNA; and PAE-nsRNA (n = 6 mice per group), and treated with systemic administration of different siRNA formulations (40 µg siRNA per mice equivalent, 150 µL of 20 µM stock) via tail vein injection every 2 days for six injections. The body weights of the mice were monitored over the experiment period. The growth of pulmonary tumors was monitored by IVIS Spectrum *in vivo* imaging system (PerkinElmer). D-luciferin (150 mg/kg, Beyotime, Shanghai, China) was intraperitoneally injected into mice 10 min before the imaging. Bioluminescence images (BLI) images of the mice were performed at day 14 and 28 post-tumor implantation. On day 28, the mice were killed and lungs were collected for *ex vivo* BLI analyses and photographed.

**PDX tumor model**

Balb/c nude mice were used for the establishment of the HCC PDX model. Briefly, we collected the primary HCC tissues (P1) from two patients after surgical resection and kept the specimens in iced culture medium supplemented with 1% penicillin/streptomycin. Then, the tissues were trimmed and diced into 2-3 mm^3^ fragments and subcutaneously implanted into the flanks of Balb/c nude mice. When the xenografted tumors grew up to 1-2 cm^3^, we harvested the tissues from the mice bearing PDX tumors (P2) and cut them into pieces. The tumor fragments were further implanted into Balb/c nude mice for the serial transplantation. When the tumor volume reached 50 mm^3^, the mice (P3) were randomized into different groups: Saline; Free siRNA; PAE-siRNA; and PAE-nsRNA (n = 6 mice per group), and treated with different siRNA formulations (40 µg siRNA per mouse equivalent, 150 µL of 20 µM stock) via tail vein injection every 2 days for six injections. On day 48, the mice were killed, tumor xenografts were excised, weighted and imaged.

**Orthotopic tumor model**

We created orthotopic tumors in Balb/c nude mice to mimic the natural liver cancer microenvironment. Briefly, mice were anaesthetized and a midline incision was made to expose the liver. 1×10^6^ HepG2 cells were injected into the lower surface of the left liver lob of Balb/c nude mice using a micro-syringe (Hamilton). At the puncture site, hemostasis is stopped by compression and electrocoagulation with hemostatic pen. Then the liver and abdomen were closed with silk sutures. Ten days after the cell injection, the mice were randomly divided into different groups: saline; free siRNA; PAE-siRNA; and PAE-nsRNA (n = 6 mice per group), and treated with systemic administration of different siRNA formulations (40 µg siRNA per mouse equivalent, 150 µL of 20 µM stock) via tail vein injection every 2 days for six injections. The body weights of the mice were monitored over the experiment period. On day 22, the mice were performed euthanasia, their livers were excised and imaged.

**Whole-body imaging of mice with PET and CT (PET/CT) scans**

PET/CT scans were performed in Balb/c nude mice 20 days after transplantation. The production of ^18^F-FDG was carried out according to a method described elsewhere. After intravenous injection of ^18^F-FDG, the mice were placed in an imaging chamber and kept under anesthesia. The kinetics and distribution patterns of the radiolabeled compound were determined with a small animal PET scanner (Siemens Biograph 64HD PET/CT, Siemens, Germany). The total scan time for each scan is 2 min. Regions of interest (ROI) were manually drawn over the liver tumors. Tracer uptake by the liver tumors were quantified by maximum standardized uptake values (SUV max) using the formula: SUV max = maximum tissue activity concentration (μCi/ml)/injected dose (μCi) × body weight (g).

***In vivo* toxicity**

For histology analysis, 24 h after the last intravenous injection of drugs, major organs (heart, liver, spleen, lung and kidney) and tumor tissues of the mice were fixed, sectioned and stained with hematoxylin and eosin (H&E). The slices were observed by microscope (Leica DMI 6000B). At 24 h after the last intravenous injection of drugs, blood was drawn from the venous plexus of the eyes of the mice. Blood samples were immediately centrifuged at 3,000g for 5 min at 4 °C, and the supernatant was collected for hematological analysis. For blood biochemical analysis, ALT, AST, blood urea nitrogen and creatinine values were measured using Modular analytics (Roche, Germany), as indicators of hepatic (ALT and AST) and renal (blood urea nitrogen and creatinine) functions.

**TUNEL, Ki-67 and immunofluorescence (****IF) staining**

The analysis of apoptosis *in vivo* was performed by TUNEL staining based on the standard scheme. The paraffin-embedded tissue samples were separated and the antigens were recovered for Ki-67 detection according to the manufacturer’s test kit instructions. As for IF staining, the paraffin-embedded tumor tissue was deparaffinized for antigen repair. IF staining was done with anti-ATG16L1, Bax, Bcl-2, and Caspase-3 according to the standard protocol. The nucleus was stained with DAPI, followed by the use of fluorescent microscope to obtain images.

**References**

1 Zeng, K. *et al.* CircHIPK3 promotes colorectal cancer growth and metastasis by sponging miR-7. *Cell Death Dis* **9**, 417, doi:10.1038/s41419-018-0454-8 (2018).

2 Zhao, Z. *et al.* CircSOD2 induced epigenetic alteration drives hepatocellular carcinoma progression through activating JAK2/STAT3 signaling pathway. *J Exp Clin Cancer Res* **39**, 259, doi:10.1186/s13046-020-01769-7 (2020).

3 Peng, L. *et al.* circCUL2 regulates gastric cancer malignant transformation and cisplatin resistance by modulating autophagy activation via miR-142-3p/ROCK2. *Mol Cancer* **19**, 156, doi:10.1186/s12943-020-01270-x (2020).

4 Cheng, Z. *et al.* circTP63 functions as a ceRNA to promote lung squamous cell carcinoma progression by upregulating FOXM1. *Nat Commun* **10**, 3200, doi:10.1038/s41467-019-11162-4 (2019).

5 Wang, K. *et al.* A circular RNA protects the heart from pathological hypertrophy and heart failure by targeting miR-223. *Eur Heart J* **37**, 2602-2611, doi:10.1093/eurheartj/ehv713 (2016).

6 Li, B. *et al.* circNDUFB2 inhibits non-small cell lung cancer progression via destabilizing IGF2BPs and activating anti-tumor immunity. *Nat Commun* **12**, 295, doi:10.1038/s41467-020-20527-z (2021).

**Supplementary Table 1. Clinical information of 10 HCC patient samples used for RNA-sequencing.**

| **Patient ID** | **Gender** | **Age** | **HBV-DNA (IU/ml)** | **Serum AFP (ng/ml)** | **Maximal tumor diameter (cm)** | **Number of tumors** | **Portal invasion** | **Cirrhosis** | **HCC stage** | |
| --- | --- | --- | --- | --- | --- | --- | --- | --- | --- | --- |
|  |  |  |  |  |  |  |  |  | **TNM** | **BCLC** |
| 1232 | Male | 50 | 1.90×10^4^ | 11.20 | 4 | 1 | No | Yes | T1N0M0 | A |
| 1263 | Male | 60 | 1.20×10^6^ | 14.04 | 5 | 1 | No | Yes | T1N0M0 | A |
| 1302 | Male | 55 | <500 | 2.11 | 3.5 | 1 | No | Yes | T1N0M0 | A |
| 1329 | Male | 44 | 1.73×10^2^ | 8.23 | 3 | 1 | No | Yes | T1N0M0 | A |
| 1340 | Male | 49 | 7.40×10^2^ | 106.70 | 2.2 | 1 | No | Yes | T1N0M0 | A |
| 1495 | Male | 57 | <500 | 9.47 | 6 | 1 | No | Yes | T1N0M0 | A |
| 1497 | Male | 57 | 6.20×10^5^ | 17.70 | 7 | 1 | Yes | Yes | T2N0M0 | C |
| 1517 | Female | 43 | 6.50×10^5^ | 73.18 | 5.5 | 1 | No | Yes | T1N0M0 | A |
| 1522 | Male | 69 | <500 | 4.26 | 4.5 | 1 | No | Yes | T1N0M0 | A |
| 1559 | Male | 58 | <500 | 30.46 | 2.3 | 1 | No | Yes | T1N0M0 | A |

**Supplementary Table 2. Clinical information of 35 HCC patients’ samples for validation of circMDK expression.**

| **Patient number** | **Gender** | **Age** | **HBV-DNA (IU/ml)** | **Serum AFP (ng/ml)** | **Maximal tumor diameter (cm)** | **Number of tumors** | **Portal invasion** | **Cirrhosis** | **HCC stage** | |
| --- | --- | --- | --- | --- | --- | --- | --- | --- | --- | --- |
|  |  |  |  |  |  |  |  |  | **TNM** | **BCLC** |
| 1 | Male | 30 | 4.92E+07 | 271.4 | 7 | 1 | No | Yes | T1N0M0 | A |
| 2 | Male | 47 | ＜5E+02 | 1557 | 14.7 | 2 | Yes | Yes | T4N0M1 | D |
| 3 | Male | 62 | ＜5E+02 | 8.12 | 3.8 | 1 | No | Yes | T3N0M0 | C |
| 4 | Male | 57 | 6.53E+07 | 35774 | 10 | 1 | No | Yes | T2N0M0 | C |
| 5 | Female | 27 | 5.46E+06 | 48400 | 10 | 5 | Yes | Yes | T4N1M1 | D |
| 6 | Male | 69 | ＜5E+02 | 66.7 | 5 | 1 | Yes | No | T3bN1M1 | D |
| 7 | Male | 48 | ＜5E+02 | 4.39 | 3.0 | 1 | Yes | Yes | T1N0M0 | A |
| 8 | Male | 48 | 7.67E+02 | 141 | 4.0 | 1 | Yes | Yes | T2N0M0 | C |
| 9 | Male | 50 | ＜5E+02 | 8.68 | 9.5 | 1 | Yes | No | T3N0M1 | D |
| 10 | Female | 64 | 3.78E+4 | 3.51 | 3.0 | 2 | No | Yes | T1N0M0 | A |
| 11 | Male | 41 | ＜5E+02 | 374665 | 4.0 | 1 | Yes | No | T1N0M0 | A |
| 12 | Female | 62 | 2.42E+06 | 9.28 | 3.8 | 1 | No | Yes | T1N0M0 | A |
| 13 | Male | 54 | 1.48E+04 | 3271 | 7.0 | 2 | No | Yes | T2N1M0 | C |
| 14 | Female | 60 | ＜5E+02 | 2620 | 4.0 | 1 | Yes | Yes | T3aN1M1 | D |
| 15 | Female | 63 | ＜5E+02 | 47323 | 7.4 | 2 | No | Yes | T1N0M0 | A |
| 16 | Male | 79 | ＜5E+02 | 32056 | 10 | 1 | Yes | Yes | T1N0M0 | A |
| 17 | Male | 63 | 5.28E+04 | 12.55 | 1.1 | 1 | No | Yes | T1N0M0 | A |
| 18 | Female | 43 | ＜5E+02 | 484000 | 22.1 | 3 | Yes | Yes | T1N0M0 | A |
| 19 | Male | 61 | ＜5E+02 | 8.13 | 3.4 | 1 | No | Yes | T3bN1M1 | D |
| 20 | Male | 71 | 3.67E+04 | 9.62 | 2.2 | 1 | Yes | Yes | T1N0M0 | A |
| 21 | Male | 50 | ＜5E+02 | 401 | 4.8 | 1 | No | Yes | T4N1M1 | D |
| 22 | Male | 65 | 5.32E+05 | 5.63 | 10 | 2 | Yes | No | T1N0M0 | A |
| 23 | Female | 63 | 6.20E+05 | 2646 | 3.0 | 1 | No | Yes | T4N1M1 | D |
| 24 | Male | 52 | 1.07E+05 | 3530 | 13.6 | 1 | No | Yes | T2N0M0 | C |
| 25 | Male | 82 | ＜5E+02 | 15883 | 3 | 2 | Yes | Yes | T2N0M0 | C |
| 26 | Male | 57 | 7.94E+02 | 2629 | 21.6 | 1 | Yes | Yes | T2N1M0 | C |
| 27 | Male | 65 | 1.71E+04 | 23406 | 4.2 | 1 | No | Yes | T1N0M0 | A |
| 28 | Female | 45 | 5.32E+06 | 966 | 2.2 | 1 | No | Yes | T2N0M0 | C |
| 29 | Male | 48 | 4.32E+05 | 44.5 | 20.9 | 1 | Yes | No | T2N1M1 | D |
| 30 | Male | 28 | 6.02E+03 | 889 | 14.1 | 1 | Yes | Yes | T3bN0M1 | D |
| 31 | Female | 55 | 7.74E+03 | 24.5 | 1.6 | 1 | Yes | Yes | T4N1M0 | D |
| 32 | Female | 64 | ＜5E+02 | 6.28 | 1.0 | 1 | No | Yes | T1N0M0 | A |
| 33 | Male | 80 | ＜5E+02 | 7.28 | 3.0 | 2 | Yes | Yes | T1N0M0 | A |
| 34 | Male | 48 | 4.75E+03 | 1855 | 4.7 | 1 | Yes | Yes | T4N1M1 | D |
| 35 | Female | 20 | 1.96E+04 | 201238 | 32.8 | 3 | Yes | No | T4N0M1 | D |

**Supplementary Table 3. Associations between the expression levels of circMDK and the clinicopathological characteristics of 35 HCC patients.**

| **Clinical characteristics** | | **CircMDK expression** | | **χ^2^-value** | ***p*-value** |
| --- | --- | --- | --- | --- | --- |
|  |  | **High (n=18)** | **Low (n=17)** |  |  |
| Sex | |  |  |  |  |
| Male | | 13 | 11 | 0.349 | 0.555 |
| Female | | 5 | 6 |  |  |
| Age, years | | |  |  |  |
| <60 | | 8 | 12 | 2.289 | 0.130 |
| ≥60 | | 10 | 5 |  |  |
| AFP, ng/mL | | |  |  |  |
| <40 | | 4 | 6 | 0.412 | 0.521 |
| ≥400 | | 14 | 11 |  |  |
| HBV DNA, cps/mL | |  |  |  |  |
| <500 | | 10 | 5 | 2.307 | 0.129 |
| ≥500 | | 8 | 12 |  |  |
| Cirrhosis | |  |  |  |  |
| Absent | | 15 | 14 | 0.114 | 0.735 |
| Present | | 3 | 3 |  |  |
| Microvascular invasion | | |  |  |  |
| Absent | 8 | | 12 | 2.289 | 0.130 |
| Present | 10 | | 5 |  |  |
| Tumor size, cm |  | |  |  |  |
| <5 | 8 | | 11 | 1.391 | 0.238 |
| ≥5 | 10 | | 6 |  |  |
| Tumor number | | |  |  |  |
| Single | 11 | | 14 | 0.019 | 0.890 |
| Multiple | 7 | | 3 |  |  |
| Tumor metastasis | | |  |  |  |
| Absent | 15 | | 14 | 0.114 | 0.735 |
| Present | 3 | | 3 |  |  |
| TNM | | |  |  |  |
| I-II | 6 | | 13 | 6.415 | 0.011^*^ |
| III-IV | 12 | | 4 |  |  |
| Tumor differentiation |  | |  |  |  |
| I-II | 5 | | 8 | 0.846 | 0.358 |
| III-IV | 13 | | 9 |  |  |

**χ^2^** test was used to test the association between two categorical variables.

AFP, alpha-fetoprotein.

* Statistically significant.

**Supplementary Table 4. Primers, siRNA, shRNA sequences and antibodies.**

| **Primer name** | **Application** | **qPCR primer sequence (5’→3’)** |
| --- | --- | --- |
| MDK-F | qRT-PCR | GAAGAAGGAGTTTGGAGCCGACTG |
| MDK-R | qRT-PCR | GTCTCCTGGCACTGAGCATTGTAG |
| circMDK-F | qRT-PCR | GCCTGGGAGAGGGAGGGCG |
| circMDK-R | qRT-PCR | CCGAGATGTGACCCACCAGTGCC |
| ATG16L1-F | qRT-PCR | CAAGCCGAATCTGGACTGTGGATG |
| ATG16L1-R | qRT-PCR | CGGTCGTGACTTCCTGAGACAATC |
| IGF2BP1-F | qRT-PCR | AGGCAGGCTGACGAGGTTCC |
| IGF2BP1-R | qRT-PCR | GGTTCCGTCCTTCCTTGCCAATG |
| IGF2BP2-F | qRT-PCR | CATCATCGGAAAGGAGGGCTTGAC |
| IGF2BP2-R | qRT-PCR | GCATGGATGGTGACAGGCTTCTC |
| IGF2BP3-F | qRT-PCR | TCACTTCTATGCTTGCCAGGTTGC |
| IGF2BP3-R | qRT-PCR | CCTTCTGTTGTTGGTGCTGCTTTAC |
| Fto-F | qRT-PCR | TCAACTGGAAGCACTGTGGAAGAAG |
| Fto-R | qRT-PCR | CGAGGCAAGGATGGCAGTCAAG |
| Mettl14-F | qRT-PCR | ACCAAAATCGCCTCCTCCCAAATC |
| Mettl14-R | qRT-PCR | AGCCACCTCTTTCTCCTCGGAAG |
| Mettl3-F | qRT-PCR | CTGTGTCCATCTGTCTTGCCATCTC |
| Mettl3-R | qRT-PCR | ACCTCGCTTTACCTCAATCAACTCC |
| GAPDH-F | qRT-PCR | GAAGGTGAAGGTCGGAGTC |
| GAPDH-R | qRT-PCR | GAAGATGGTGATGGGATTTC |
| β-actin-F | qRT-PCR | GGGAAATCGTGCGTGACATTAAG |
| β-actin-R | qRT-PCR | TGTGTTGGCGTACAGGTCTTTG |
| U6-F | qRT-PCR | CGCTTCGGCAGCACATATACTA |
| U6-R | qRT-PCR | CGCTTCACGAATTTGCGTGTCA |
| si-circMDK#1 | siRNAs | GGGCCAGGCCCCATAGGGA |
| si-circMDK#2 | siRNAs | CCAGGCCCCATAGGGACAA |
| si-circMDK#3 | siRNAs | GCCCCATAGGGACAAGGCA |
| si-ATG16L1#1 | siRNAs | GGATCCAGTTGCAATGATA |
| si-ATG16L1#2 | siRNAs | GTTCAAGGGTTCCCTATCT |
| siRNA control | siRNAs | UUCUCCGAACGUGUCACGU |
| has-miR-346 mimics | miRNA | AAGCTTATGTCTGCCCGCATG |
| has-miR-346 inhibitors | miRNA | CATGCGGGCAGACATAAGCTT |
| has-miR-874-5p mimics | miRNA | CTATACGGCCCCACGCAC |
| has-miR-874-5p inhibitors | miRNA | GTGCGTGGGGCCGTATAG |
| mimics NC | miRNA | UUUGUACUACACAAAAGUACUG |
| inhibitors NC | miRNA | CAGUACUUUUGUGUAGUACAAA |
| circMDK Probe | pulldown | GCCCUGCCUUGUCCCUAUGGGGCCUGGCCCACGCCCUCCCUC |
| Control probe | pulldown | UUGUACUACACAAAAGUACUG |

**Supplementary Table 5. Blood biochemistry of tumor-bearing mice treated with saline, PAE-nsRNA and PAE-siRNA.**

|  | **Saline** | **PAE-nsRNA** | **PAE-siRNA** |
| --- | --- | --- | --- |
| WBC (10^9^/L) | 6.16±0.33 | 5.86±0.24 | 6.16±0.26 |
| RBC (10^12^/L) | 8.59±0.22 | 8.33±0.38 | 8.53±0.32 |
| HGB (g/L) | 91.27±3.82 | 87.7±4.05 | 93.76±0.95 |
| PLT (10^9^ /L) | 692.56±24.36 | 686.0±25.18 | 757.43±9.53 |
| BUN (mg/dL) | 7.79±0.34 | 8.32±0.34 | 8.53±0.3 |
| CRE (mg/dL) | 88.73±2.17 | 84.33±3.20 | 84.53±1.55 |
| AST (U/L) | 48.0±2.36 | 49.33±1.34 | 47.56±2.73 |
| ALT (U/L) | 26.93±0.57 | 25.33±0.75 | 24.3±0.37 |

Data are expressed as the mean + SD (*n* = 3).

WBC, white Blood Cells; RBC, red blood cells; HGB, hemoglobin; PLT, platelets; BUN, blood urea nitrogen; CRE, creatinine; AST, glutamic oxalacetic transaminase; ALT, glutamic-pyruvic transaminase.

**Abbreviations:** AFP, alpha-fetoprotein; ANL, adjacent noncancerous liver; CCK-8, cell Counting Kit-8; cDNA, complementary DNA; FBS, fetal bovine serum; FISH, fluorescence in situ hybridization; gDNA, genomic DNA; H&E, hematoxylin and eosin; IF, immunofluorescence; PDX, patient-derived xenograft; PI, propidium iodide; rRNA, ribosomal RNA; RIP, RNA-binding protein immunoprecipitation; ROI, Regions of interest; SUV max, maximum standardized uptake values; TNM, tumor-node-metastasis.

**Supplementary Figure Legends**

**Figure S1 The correlation characterization of circMDK, MDK expression, and prognosis in HCC.** (A) Volcano plot of the differences in circRNA abundance between HCC tumor tissues and matched peritumor tissues. Cut off is Log2 (fold change) > 1.0 or < -1.0, *p* value *<* 0.05*.* (B) Preliminary Kaplan-Meier analysis for the correlation between circMDK expression and overall survival in 35 HCC patients. (C) The expression of MDK in human HCC tissue and normal liver tissue. (D) Kaplan-Meier analysis revealed the prognostic values of MDK. (E) Analysis for RNA levels of circMDK and MDK after treatment with RNase R in Huh7 cells. ****p <* 0.001. (F) Levels of circMDK in the nuclear and cytoplasmic fractions of Huh7 cells. ****p <* 0.001. (G) Fluorescence in situ hybridization (FISH) assay was conducted to determine the subcellular localization of circMDK. Scale bars are 5 μm. (H) Expression levels of circMDK in Huh7 and Hep3B cells treated with different siRNA of circMDK (#1, 2,3). **p <* 0.05; ***p <* 0.01 (I) Analysis for RNA levels of circMDK and MDK after treatment with different siRNA#1 of circMDK. ***p <* 0.01. Data are shown as mean ± SD of three independent experiments.

**Figure S2 The binding site of miR-346 and miR-874-3p with circMDK.** (A) Schematic illustration exhibiting overlapping of the target miRNAs of circMDK predicted by miRDB, miRanda and targetscan. (B) The predicted binding sites of miR-346 and miR-874-3p in the 3’-UTR of circMDK. The red nucleotides represent mutant sequences of target sites. (C) FISH assay showed that circMDK colocalized with miR-346 and miR-874-3p in the cytoplasm of Hep3B cells. Scale bars are 5 μm. (D) The predicted binding sites of miR-346 and miR-874-3p in the 3’-UTR of ATG16L1. The red nucleotides represent mutant sequences of target sites.

**Figure S3 MiR-346** **and** **miR-874-3p are responsible for circMDK-mediated proliferation, migration, invasion and apoptosis.** (A) Cell proliferation assay for HCC cells co-transfected with ex-circMDK together with miR-346 or miR-874-3p mimic. ***p <* 0.01; ****p <* 0.001. (B) Representative images (left) and quantification (right) of the colony formation assay in HCC cells co-transfected with the above-mentioned plasmids. **p <* 0.05; ***p <* 0.01. (C) Representative images and quantification of transwell migration and (D) invasion assays in HCC cells co-transfected with the above-mentioned plasmids. Scale bars are 200 µm. (E) Representative images (left) and quantification (right) of apoptosis assays in HCC cells co-transfected with the above-mentioned plasmids. **p <* 0.05; ***p <* 0.01. Data are shown as mean ± SD of three independent experiments.

**Figure S4 Characterization and effects of knockdown of** **circMDK with PAE-siRNA complex *in vitro*.** (A) Scheme of the preparation of PAE-siRNA complex. The negatively charged siRNA was absorbed into cationic PAEs by electrostatic interaction. (B) Stability of free siRNA and PAE-siRNA against serum nucleases. (C) Cellular uptake of free siRNA and PAE-siRNA into HepG2 cells. SiRNA was labelled with Cy5 dye (Cy5-siRNA; red), and nuclei were counterstained with 4,6-diamidino-2-phenylindole (DAPI; blue). Scale bars were 5 µm. (D) Cytotoxicity of PAE-siRNA by CCK-8 assays after incubated with HepG2 cells for 24 h with the siRNA concentration of 0 to 5,000 nM compares with Lipofectamine 3000 transfection agent (Lipo3000-siRNA). (E) Expression of circMDK mRNA in HepG2 cells analyzed by qRT-PCR. ***p* < 0.01. (F) The proliferation capacity of HepG2 cells treated with PAE-siRNA or PAE-nsRNA (100 nM siRNA equivalent) within 4 days was measured by CCK-8 assay. **p < 0.01. (G) Representative images and quantification of colony formation in HepG2 cells after receiving different treatments for 14 days. ***p* < 0.01. (H) The migratory and invasive potential of HepG2 cells treated with various nanoparticles was performed by transwell assay. ***p* < 0.01. Scale bars are 200 µm. Data are presented as mean ± SD (n = 3).

**Figure S5 The antitumor effects of PAE-siRNA complex in subcutaneous hepatic tumors.** (A) Schematic illustration of HepG2 subcutaneous model establishment and treatment. After the formation of subcutaneous tumors, Balb/c nude mice were treated with peritumoral (left panel) and intravenous (right panel) injection, respectively. In the left panel, the mice were divided into four groups including saline group, Free siRNA group, PAE-siRNA group and PAE-nsRNA group (n = 6). In the right panel, the mice were divided into three groups including saline group, Free Cy5-siRNA group and PAE-Cy5-siRNA group (n = 6). (B) Body weight change of mice during anticancer treatment. Data are shown as means ± SD (n = 6). (C) Tumor growth curve after the indicated treatments. ***p <* 0.01. (D) Photographs of the *ex vivo* tumors and (E) tumor weight from each group at the end of the various treatments. Scale bar is 1cm. ***p <* 0.01. (F) Representative images (left) and quantification (right) of tumor sections stained with H&E. **p* < 0.05; ***p* < 0.01. (G) Representative images (left) and quantification (right) of tumor sections stained with Ki-67. ***p* < 0.01. (H) Representative images (left) and quantification (right) of IF of ATG16L1. ***p* < 0.01.

**Figure S6** **Biodistribution of PAE-siRNA complex in** **subcutaneous tumor model.** (A) Representative images (left) and quantification (right) of IF of TUNEL. **p* < 0.05; ***p* < 0.01. (B) Representative images (left) and quantification (right) of IF of Bax. ***p* < 0.01. (C) Representative images (left) and quantification (right) of IF of Bcl-2. ***p* < 0.01. (D) Representative images (left) and quantification (right) of IF of Caspase-3. ***p* < 0.01. Scale bars are 50 μm. (E) Biodistribution of PAE-siRNA complex *in vivo*. Balb/c nude mice bearing subcutaneous HepG2 tumors were treated with different Cy5-siRNA formulations (40 µg Cy5-siRNA per mouse equivalent, 150 µL of 20 µM stock) via tail vein injection. Fluorescent images of mice were obtained with *in vivo* fluorescence imaging system after different time points (6h, 12h, and 24h) (n = 6). (F) Representative images (top) and quantification (bottom) of the major organs and tumors by *ex vivo* imaging after 24 h of different Cy5-siRNA injection (n = 6). ***p <* 0.01. (G) Photographs of the *ex vivo* tumors and (H) tumor weight from each group at the end of the various treatments. Scale bar is 1cm. ***p <* 0.01.

**Figure S7 Antitumor effects of** **PAE-siRNA complex in metastatic tumor model.** (A) Schematic illustration of HepG2-luc cells pulmonary metastasis model establishment and treatment. (B) Changes of mouse body weight during anticancer treatment. Data are shown as means ± SD (n = 6). (C) Representative images of bioluminescence images from each group after intraperitoneally injected D-luciferin. (n = 3). (D) Representative images of metastatic lung nodules. (E) Representative bioluminescence images from each group (n = 3). (F) Representative images (left) and quantification (right) of H&E staining of lung from each group. **p* < 0.05; ***p* < 0.01. Scale bars are 100 µm (top) and 200 µm (bottom).

**Figure S8 Validation diagrams for PDX model construction and Histological observation of tissue sections.** (A) Representative images (top) and quantification (bottom) of H&E staining and (B) Representative images (top) and quantification of (bottom) Hep staining of subcutaneous tumor tissues from primary liver tumor of patients (P1) to subcutaneous tumor of mice (P2 and P3). Scale bars are 50 µm. (C) Changes of mouse body weight during anticancer treatment. Data are shown as means ± SD (n = 6). (D) H&E staining of tissue sections from major organs (heart, liver, spleen, lung and kidney) of mice was performed after the treatment. Scale bar is 100 µm.
